# Resolution of Praziquantel

**DOI:** 10.1371/journal.pntd.0001260

**Published:** 2011-09-20

**Authors:** Michael Woelfle, Jean-Paul Seerden, Jesse de Gooijer, Kees Pouwer, Piero Olliaro, Matthew H. Todd

**Affiliations:** 1 School of Chemistry, The University of Sydney, Sydney, New South Wales, Australia; 2 Syncom B.V., Groningen, The Netherlands; 3 UNICEF/UNDP/World Bank/WHO Special Programme for Research and Training in Tropical Diseases (TDR), World Health Organization, Geneva, Switzerland; McGill University, Canada

## Abstract

**Background:**

Praziquantel remains the drug of choice for the worldwide treatment and control of schistosomiasis. The drug is synthesized and administered as a racemate. Use of the pure active enantiomer would be desirable since the inactive enantiomer is associated with side effects and is responsible for the extremely bitter taste of the pill.

**Methodology/Principal Findings:**

We have identified two resolution approaches toward the production of praziquantel as a single enantiomer. One approach starts with commercially available praziquantel and involves a hydrolysis to an intermediate amine, which is resolved with a derivative of tartaric acid. This method was discovered through an open collaboration on the internet. The second method, identified by a contract research organisation, employs a different intermediate that may be resolved with tartaric acid itself.

**Conclusions/Significance:**

Both resolution procedures identified show promise for the large-scale, economically viable production of praziquantel as a single enantiomer for a low price. Additionally, they may be employed by laboratories for the production of smaller amounts of enantiopure drug for research purposes that should be useful in, for example, elucidation of the drug's mechanism of action.

## Author Summary

The drug praziquantel (PZQ) is used very widely in both animal and human medicine, where it is the mainstay of the treatment of the neglected tropical disease schistosomiasis. The drug is currently manufactured and administered as a racemate (1∶1 mixture of enantiomers) but for various reasons the large-scale production of PZQ as the single active enantiomer is very desirable. We describe here the preparation of praziquantel as a single enantiomer using classical resolution. The protocols are experimentally simple and inexpensive. One method was found and validated by an unusual research mechanism—open science—where the details of the collaboration (involving academic and industrial partners) and all research data were available on the web as they were acquired, and anyone could participate. The other route was found in parallel by a contract research organisation. Besides being possible routes by which praziquantel may be produced in large quantities for the affected communities, it is also hoped that these methods can be used for the production of smaller quantities of enantiopure PZQ for pharmacological studies.

## Introduction

Schistosomiasis (bilharziosis) is termed a "neglected" tropical disease owing to the continuing low level of investment in treatments, prevention and research, yet the disease accounts for an extraordinarily high level of suffering around the world.[1], [2] Schistosomiasis has been called a "silent pandemic".[3]

Over the past decades several compounds have been used for the treatment of schistosomiasis,[4]–[6] but today there is only one drug of choice, a highly effective small molecule called praziquantel (PZQ).[7], [8] PZQ is produced on a very large scale (300 metric tons worth of API per year) and is used primarily in veterinary medicine. In human medicine, PZQ is used essentially as preventive (mass) chemotherapy for all forms of schistosomiasis - whereby school-aged children or entire communities are given a dose of PZQ once a year. Such mass treatment programs (e.g. that coordinated by the Schistosomiasis Control Initiative)[9] deploy 100 million tablets annually, and there is expected to be a further large growth in the demand for PZQ in the coming years.[10]

With increasing use comes an increased risk of the development of resistance or tolerance by the parasite. Decreased drug sensitivity was developed via an artificial selection experiment in the laboratory,[11] and reports of similar decreases have already been noted in the field.[12]–[15] Reliance on a single drug for intensive mass treatment is risky. While there have been attempts to find bioactive PZQ analogs,[16]–[18] as well as the discovery of new compounds for the treatment of schistosomiasis based on different modes of action,[19], [20] in the short term it is sensible to continue to use PZQ in a way that maximizes its life as a useful drug.

PZQ is synthesized and administered as a racemate. The L-(–)-enantiomer is the eutomer[21]–[24] and has the (*R*) configuration.[7], [23] Administration of the pure eutomer resulted in fewer side effects than the racemate.[22] The inactive (+)-enantiomer is associated with side effects and is also primarily responsible for the extremely bitter taste of the pill;[25] factors such as taste and large pill size contribute to there being a compliance problem with PZQ in the affected communities.[26]–[27] The typical dose per pill (40 mg kg^−1^, pill contains 600 mg active pharmaceutical ingredient (API)) is large. The pill is difficult to swallow for children (who are the main target of mass chemotherapy campaigns) often requiring tablets to be split and crushed, which brings out the bitter taste even further. Decreasing the pill size, reducing side effects and removing the bitter taste, while having the right amount of the active ingredient, could be accomplished were the drug to be made available as a single enantiomer. For these reasons investigations into the viability of a process-scale route to enantiopure PZQ were included in the WHO/TDR business plan for 2008–2013.[28] Availability of the separate enantiomers would be a valuable tool for the elucidation of the mechanism of action of the drug, still unknown after more than 30 years of use;[29] in such experiments the inactive enantiomer would act as the perfect control.

**Figure 1:**
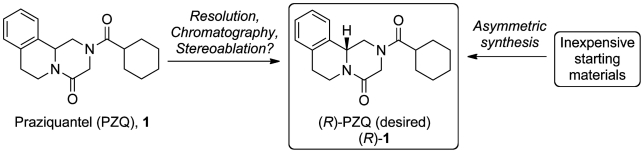
General approaches to the preparation of enantiopure praziquantel (PZQ).

There are typically four methods available for the conversion of a racemic synthesis to one that generates a single enantiomer (Figure 1): 1) Enantioselective synthesis, 2) Chromatographic separation, 3) Stereoablation (destruction and selective reconstruction of the stereocenter) and 4) Resolution. To date, reports of the preparation of enantiopure PZQ either have insufficient detail to allow for their appraisal, or likely do not have the potential for the large-scale production of the drug given the severe price constraint;[25], [30]–[34] for example with enantioselective chromatographic approaches significant quantities of solvents would be required.

Racemic praziquantel is off-patent. Through market forces, and its production on such a large scale, the racemate is available for approximately US10¢ per gram. A reasonable price for wide distribution of the enantiopure compound would therefore be approximately US20¢ per gram since half the dose is required of the enantiopure compound. Can enantiopure PZQ be obtained without a significant increase in price? It is a challenge for either academia or industry to solve this problem, for different reasons. This article describes the discovery of two solutions, one by an open collaborative project that starts from *rac*-PZQ, and one conducted by a contract research organization that employs a commercially-available precursor. We term these “resolutions of praziquantel” for simplicity. Formally both processes involve the resolution of a precursor or derivative that is then converted to praziquantel.

## Methods

(*rac*)-PZQ was a gift from WHO/TDR; the sample was originally synthesized by Merck. Analytical samples of (*R*)- and (*S*)-PZQ were a gift from Intervet Innovation GmbH and were prepared according to the literature method.[25]

### Hydrolysis of PZQ

#### HPLC Analysis of (*rac*)-PZQ

Enantiomeric composition of praziquantel can be assayed by a number of enantioselective HPLC columns. For example: Chiralcel OD-H column, hexane/isopropanol/triethylamine solvent system (60∶40∶0.1), flow rate 0.7 mL per minute. Retention times: 11.6 (*R*-(–)-PZQ) and 13.7 (*S*-(+)-PZQ) minutes.[35] (Figure S1)

#### HPLC Analysis of (*rac*)-PZQamine

Enantioselective HPLC columns suitable for baseline separation of the enantiomers of praziquanamine include Chiralcel OJ-H, Chiralpak IA and AS-H;[36] (Text S1) eluent solvent system: heptane/EtOH/Et_2_NH (60∶40∶0.2) at 0.5–0.7 mL/min flow rate. Columns found to be unsuitable include Chiralcel OD,[17] OD-H, Chiralpak IB[36] and AD-H.[37] (Figure S2).

#### Hydrolysis of (*rac*)-PZQ to (*rac*)-PZQamine

[38] (*rac*)-PZQ (20.0 g, 64.0 mmol) was dissolved in a mixture of EtOH (150 mL) and 1 N HCl (600 mL) and heated at reflux for 26 h. The solution was cooled to rt and washed with ethyl acetate (3×10 mL). The ice-cooled solution was adjusted to pH 12 with 5 N NaOH and extracted with DCM (4×30 mL). The combined organic layers were washed with brine, dried over sodium sulfate and concentrated under reduced pressure. The residual yellow solid (12.4 g, 61.3 mmol, 96%) was recrystallized from toluene and a further batch of analytically identical crystals was obtained from the mother liquor after concentration and recrystallization. PZQamine was thus obtained as a pale yellow solid (11.9 g, 58.8 mmol, 92% yield).

m.p. 117–118°C (lit.[17] 116–118°C and[39] 117–119°C). ^1^H NMR (CDCl_3_, 200 MHz): δ 1.76 (bs, 1H), 2.64–3.02 (m, 4H), 3.49 (d, J = 17.6, 1H), 3.61 (d, J = 17.4, 1H), 3.67 (ddd, J = 13.2, 4.7, 1.2, 1H), 4.69–4.85 (m, 2 H), 7.04–7.20 (m, 4H) (Figure S3). ^13^C NMR (CDCl_3_, 50.3 MHz): d = 29.4, 39.4, 50.4, 50.7, 57.5, 125.3, 127.2, 127.6, 130.0, 134.8, 135.6, 167.9. (Figure S4) Spectroscopic data match those reported in the literature.[17], [39]

#### Hydrolysis of (*S*)-PZQ

[40] To (*S*)-PZQ (300 mg, 960 µmol) in EtOH (3 mL) was added 1 N HCl (12 mL) and the mixture was heated at reflux for 18 h. The yellow solution was cooled to rt and adjusted to pH 12 by addition of aq. 5 N NaOH. The solution was extracted with DCM (3×5 mL), the combined organic layers were washed with brine, dried over NaSO_4_ and concentrated under reduced pressure. The remaining yellow solid was purified by flash column chromatography (silica gel, ethyl acetate∶methanol∶triethylamine, 4∶1∶0.01) to give (*S*)-(+)-PZQamine as a pale yellow solid (122 mg, 63%). m.p. 117–118°C. (lit.[32] 120°C). *R*_f_ 0.23 (ethyl acetate∶methanol∶triethylamine, 4∶1∶0.01). [α]_D_^20^ = 296° (c = 1, DCM) (lit.[32] [α]_D_^20^ ((*R*)-(–)-PZQamine)  = -306° (solvent, concentration and temperature not specified; another source[17] quotes (*R*)-PZQamine and (*S*)-PZQamine as [α]_D_^20^ = –152 (c = 4.0×10^–3^, CH_2_Cl_2_) and [α]_D_^20^ = +146 (c = 3.1×10^–3^, DCM) for enantiomeric excesses higher than 95 and 91%, respectively.

### Relationship between Optical Purity and Optical Rotation for PZQ[41]

To a solution of (*R*)-(–)-PZQ (c = 1, EtOH) was added a solution of (*S*)-(+)-PZQ (c = 1, EtOH) to give a total volume of 1 mL (Table S1). The results indicate a linear relationship between optical purity and optical rotation (Figure S5).

### Relationship between Optical Purity and Optical Rotation for PZQamine[42]

To a solution of (*S*)-(+)-PZQamine (c = 1, DCM) was added a solution of *rac*-PZQamine (c = 1, DCM) to give a total volume of 1 mL (Table S2). The results indicate a linear relationship between optical purity and optical rotation (Figure S6).

### Synthesis of Resolving Agents

#### (–)-Dibenzoyl-*L*-tartaric Acid

[43] A mixture of L-(+)-tartaric acid (27.0 g, 180 mmol) and benzoyl chloride (73 mL, 630 mmol) was heated at 130°C for 4 h. The reaction mixture was allowed to cool to rt, the pale yellow solid was filtered, washed with cold diethyl ether (30 mL) and recrystallized from toluene (400 mL) to give (–)-dibenzoyl-L-tartaric acid anhydride as a colourless crystalline solid (48.3 g, 79%). The anhydride (48.3 g, 141 mmol) was dissolved in a mixture of acetone (200 mL) and water (20 mL) and heated at reflux for 2 h. Water (200 mL) was added and acetone was removed under reduced pressure. After further addition of water (200 mL) the mixture was heated at reflux for 5 min and allowed to cool to rt then cooled to 0°C and held at that temperature for 30 min. The precipitate was filtered and dried by lyophilization. (Alternatively, the solid could be dissolved in ethyl acetate, dried over sodium sulfate and concentrated under reduced pressure to give a colourless oil.) The residue (∼50 g) was recrystallized from isopropanol/hexane (500 mL, 1∶1) and the mother liquor was concentrated and recrystallized two times from isopropanol/hexane (400 mL, 1∶2 and 50 mL, 2∶1) to give (–)-dibenzoyl-L-tartaric acid•2 isopropanol as colourless spicular crystals (58.9 g, 68%).

m.p. 99–102°C (lit.[44] 95–98°C for (–)-dibenzoyl-L-tartaric acid•H_2_O). ^1^H NMR (DMSO-*d*6, 200 MHz): δ = 1.03 (d, J = 6 Hz, 12H), 3.78 (sep, J = 6 Hz, 2H), 5.88 (s, 2H), 7.57–7.64 (m, 4H), 7.70–7.74 (m, 2H), 8.00–8.04 (m, 4H), 14.00 (bs, 2H) (Figure S7). ^13^C NMR (DMSO-d6, 50.3 MHz): δ = 25.5 (4C), 62.2 (2C), 71.6 (2C), 128.6 (2C), 129.1 (4C), 129.5 (4C), 134.2 (2C), 164.8 (2C), 167.3 (2C) (Figure S8). IR (neat): ν = 3465, 2975, 1730, 1237, 1098, 938, 706 cm^−1^. MS (ESI (–)) *m/z* (%): 357 (100) [M-H]^-^. HRMS (ESI (-)) Calcd. for [C_18_H_13_O_8_]: 357.0616, found: 357.0616. HRMS (ESI (+)) Calcd. for [C_18_H_14_O_8_Na^+^]: 381.0581, found: 381.0579. C_24_H_30_O_10_: calc. C 60.24%, H 6.32%; found: C 60.27%, H 6.33%. [α]_D_^20^ = -85.0° (c = 1, EtOH) (no literature data for •2*^i^*PrOH compound).

#### (+)-Di-*p*-anisoyl-D-tartaric Acid

[45], [46] A mixture of *p*-methoxybenzoic acid (33.6 g, 253 mmol) and thionyl chloride (82.0 g, 691 mmol, 50 mL) was stirred for 1 h at rt and heated at reflux for a further 3 h. Thionyl chloride was removed by distillation and the residue was concentrated under reduced pressure. To the resulting crude *p*-methoxybenzoyl chloride (2*S*,3*S*)-D(–)-tartaric acid (12.5 g, 83.0 mmol) was added and the mixture was heated at 140°C for 1 h till a pale yellow solid was formed. The mixture was heated at 160°C for a further 1 h. The mixture was allowed to cool to 100°C and toluene (100 mL) was added, the mixture was allowed to cool to rt and a further portion of toluene (50 mL) was added. The colourless solid was filtered, rinsed with a small amount of cold toluene and dried under vacuum. The (+)-di-*p*-anisoyl-D-tartaric acid anhydride was dissolved in a mixture of acetone (150 mL) and water (10 mL) and heated at reflux for 2 h. After addition of water (100 mL) acetone was evaporated and another portion of water (100 mL) was added. The colourless precipitate was filtered and dried. To remove the remaining *p*-methoxybenzoic acid the product was heated at reflux with toluene (100 mL) for 15 min, the precipitate was filtered while the mixture was hot and washed with hot toluene. The procedure was repeated three further times to give (+)-di-*p*-anisoyl-D-tartaric acid as a colourless solid (19.9 g, 57%).

m.p. 186–188.5°C (lit.[47] 186°C). ^1^H NMR (DMSO-*d*6, 200 MHz): δ = 3.50 (bs, 2H), 3.86 (s, 6H), 5.80 (s, 2H), 7.13 (d, *J* = 8 Hz, 4H), 7.97 (d, *J* = 8 Hz, 4H), 13.80 (bs, 2H). (Figure S9) ^13^C NMR (DMSO-*d*6, 50.3 MHz): δ = 55.7 (2C), 71.2 (2C), 114.4 (4C), 120.7 (2C), 131.6 (4C), 163.8 (2C), 164.3 (2C), 167.4 (2C). (Figure S10). IR (neat): ν = 2943 cm^−1^, 1720, 1662, 1601, 1243, 1170, 1103, 1012, 847, 762, 691. MS (ESI (+)) *m/z* (%): 875 (35) [2M+K]^+^, 859 (85) [2M+Na]^+^, 441 (53) [M+Na]^+^, 435 (85), 329 (100). HRMS (ESI (+)) Calcd. for [C_20_H_18_O_10_Na]^+^: 441.0792, found: 441.0790. [α]_D_^20^ = +169° (c = 1, MeOH), (lit.[47] [α]_D_^20^ = +167° (c = 1, MeOH).

### Resolution of PZQamine [48]

*rac*-Praziquanamine (10.0 g, 49.5 mmol) and (–)-dibenzoyl-L-tartaric acid•2 *i*-PrOH (23.7 g, 49.5 mmol) were dissolved in isopropanol (450 mL) and water (90 mL) by heating the stirred mixture. The solution was allowed to cool to rt and after 2 h the colourless crystals were filtered and dried to yield the salt as pale yellow crystals (12.1 g, 44%). m.p. 145.5–147.5°C. Small-scale liberation of amine (procedure below) gave [α]_D_^20^ (liberated amine)  = -242° (c = 1, DCM), implying 79% *ee* (determined by polarimetry).

The salt was recrystallized from a mixture of isopropanol (180 mL) and water (90 mL). The crystalline precipitate was kept at 5°C for 12 h before filtration, though the crystallization is essentially complete after 2 h. This procedure gave the salt as colourless spicular crystals (10.2 g, 85% from this procedure, 37% overall). m.p. 147.3–148.5°C.

#### Liberation of (R)-PZQamine

The salt (10.2 g, 18.3 mmol) was suspended in water (100 mL) and the pH of the mixture was adjusted to 11 by addition of 2 N sodium hydroxide solution. Alternatively the salt can be dissolved in 12% aq. solution of potassium carbonate (150 mL).[45] When the salt was completely dissolved the solution was extracted with dichloromethane (4×15 mL). The combined organic layers were washed with brine, dried over sodium sulfate and concentrated under reduced pressure to give *R*-(–)-PZQamine as a colourless solid (3.32 g, 33% overall). m.p. 122–123°C. [α]_D_^20^ = -305° (c = 1, DCM), 99% *ee* (determined by polarimetry).

#### Recovery of (S)-PZQamin

The combined mother liquors of the crystallization process were concentrated under reduced pressure until isopropanol was evaporated. The remaining suspension was dissolved by adding 2 N sodium hydroxide or 12% aq. solution of potassium carbonate until a pH 10–11 was reached. The solution was extracted with dichloromethane (4×15 mL). The combined organic layers were washed with brine, dried over sodium sulfate and concentrated under reduced pressure to give enantioenriched *S*-(+)-PZQamine as a yellow solid (5.34 g, 53%). m.p. 102–104°C. [α]_D_^20^ = +170° (c = 1, DCM), 56% *ee* (determined by polarimetry).

#### Recycling of the Resolving Agent

The combined basic aq. portions from both amine liberation processes were adjusted to pH 2–3 by addition of 2 N aq. HCl immediately after the extraction of PZQamine. (Allowing the aqueous portions to stand at alkaline pH tends to result in hydrolysis of the resolving agent.) The resulting colourless precipitate was filtered, washed with cold water and dried under vacuum to give (–)-dibenzoyl-L-tartaric acid as a colourless solid (21.1 g, 89%). For further purification the solid was recrystallized from acetone/hexane (1∶2).[48]

### Synthesis of R-(–)-PZQ from R-(–)-PZQamine [49]

To an ice-cooled solution of *R*-(–)-PZQamine (3.27 g, 16.2 mmol) and triethylamine (2.45 g, 3.38 mL, 24.3 mmol, 1.5 eq.) in dichloromethane (80 mL) was added dropwise cyclohexanoyl chloride (2.62 g, 2.39 mL, 17.8 mmol, 1.1 eq.) at 0°C and stirring was continued for 14 h at rt. The solution was quenched with water (10 mL) and stirred for a further 30 min. The layers were separated and the organic layer was washed with saturated sodium carbonate solution, 0.5 N HCl solution and brine, dried over magnesium sulfate and concentrated under reduced pressure. The remaining yellow oil became solid after drying under high vacuum and storing at 5°C. The pale yellow solid was recrystallized from acetone/hexane (35 mL, 1∶1 mixture) and two further batches of analytically identical crystals were obtained from the mother liquor after concentration and recrystallization. *R*-(–)-PZQ was thus obtained as colourless crystals (4.56 g, 90%, 97% *ee*). (Figure S11) m.p. 113.5–114.5°C. [α]_D_^20^ = -136° (c = 1, EtOH).

### Resolution Procedure via Benzoyl Intermediate 3

An outline description of this procedure can be found online.[50]

## Results and Discussion

A coordination website was created on which was posted the problem of the preparation of praziquantel as a single enantiomer.[51] While suggestions were received, input was initially low. In mid-2008 the project was funded by a government/NGO consortium. The resulting raw experimental data were posted in full to an open, online electronic lab notebook (based on the open source electronic lab notebook system, Labtrove, developed by the University of Southampton, UK. [52]) Periodic updates were posted on the coordination website, and the project was popularised to increase traffic (For a description of how the open science project was conducted, see the accompanying paper [53]).

Two approaches were begun in the laboratory that have so far proved intractable. The first, a community suggestion, relied on oxidation of PZQ to an enamide, which was to be subsequently hydrogenated asymmetrically[54] (a similar approach was described in a patent, employing Raney Nickel modified with tartaric acid, giving products with low optical purities – see reference [34]); this is a strong approach owing to the highly effective use of asymmetric hydrogenation in process chemistry.[55] Through an online collaborative process, catalysts are being screened for this reduction[56] but the reaction is difficult owing to the lack of a well-placed coordinating group able to direct the metal catalyst to the double bond. The second approach was based on an asymmetric Pictet-Spengler reaction.[57] Catalysts for similar reactions are known,[58] and the relevant starting material (a peptide acetal) is an intermediate in two known syntheses of PZQ.[39], [59] Unfortunately this substrate contains an unreactive aromatic ring (i.e. lacking electron donating groups), and at the time of writing no known asymmetric catalyst has given conversion to PZQ.

The third possibility was resolution. Such an approach is widely used in the process-scale production of enantioenriched intermediates because the relevant chiral resolving agents are frequently inexpensive and/or can be recycled. Inputs to the collaborative website and elsewhere suggested this approach was more likely to lead to an economically viable solution to the problem.[60] In response, this approach was prioritized.

**Figure 2:**
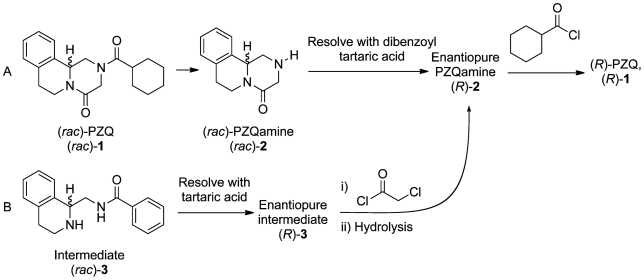
Two resolution approaches to enantiopure PZQ discovered through A) open science and B) contract research.

To effect a resolution, PZQ should be hydrolysed to praziquanamine (PZQamine, Figure 2A). The process must employ only crystallizations (rather than chromatography) to be practicable. The use of procedures that avoid the synthesis of complex catalysts, chromatographic purifications and NMR-based assessments of purity would also assist laboratories in underdeveloped countries to access enantiopure PZQ locally on smaller scales.

In the corresponding author's laboratory, PZQamine could be generated with ease, but the enantiomers could not be baseline separated by enantioselective HPLC due to a limited range of chiral stationary phases being available. This precluded a convenient local assay for resolution trials. In addition several attempts to resolve PZQamine with a range of chiral acids had met with mixed success.[61]–[62] To find a suitable chromatographic assay, an appeal for assistance was posted in several online discussion boards. In particular, the Process Chemists Group on LinkedIn furnished multiple offers of help. One company, Syncom B.V., a contract research organization in the Netherlands, additionally offered to perform a free screen of chiral acids for the resolution of PZQamine in order to discover a lead structure for the project. One gram of racemic PZQamine was shipped to Syncom. An effective chiral stationary phase was found,[36] followed by a chiral resolving agent ((–)-di-*p*-anisoyl-L-tartaric acid) that permitted the isolation of the desired (*R*)-enantiomer of PZQamine from the mother liquor in *ca.* 66% *ee*, which could be increased to 95% *ee* after one recrystallization.[63]

With this lead in hand, optimization of the process was carried out. The resolving agent in question is commercially available (reasonably expensive on a small scale) but could be synthesised from tartaric acid. However, purification away from the *p*-methoxybenzoic acid byproduct formed during its synthesis was non-trivial. It was also thought that isolation of the desired enantiomer of PZQamine from the resolved solid, rather than the mother liquor, would be more desirable; hence (+)-di-*p*-anisoyl-D-tartaric acid was prepared[46] and used for the resolution of praziquanamine to give the desired (*R*)-(–)-praziquanamine in the first-precipitated salt.[64] Such an approach is sub-optimal since this resolving agent must be obtained from unnatural enantiomer of tartaric acid. It was found that the simple expedient of using (–)-di*benzoyl*-L-tartaric acid solved both these problems, allowing the isolation of (*R*)-PZQamine in 44% yield and 80% *ee* without recrystallization and 33% yield and 97% *ee* after one recrystallization (the maximum yield for a resolution is 50%). Although we did not expect (–)-dibenzoyl-L-tartaric acid and (–)-di-*p*-anisoyl-L-tartaric acid to give opposite enantiomers of praziquanamine in the first-precipitated salts based on our experience with Dutch resolution and the “family” behaviour of resolving agents, this is not an isolated example and non-familiar behaviour has been observed in other resolutions.[65]–[66] No Horeau effect[67] is observed for either PZQ[41] or PZQamine[42] in common solvents and concentrations at room temperature, meaning that for analytically pure samples of either, optical activity can be used as an assessment of optical purity in laboratories without access to enantioselective HPLC.

(*R*)-PZQamine can be converted to (*R*)-PZQ with commercially-available cyclohexanoyl chloride in 90% yield,[49] thus completing the formal resolution of PZQ. The resolving agent can be recycled in 89% yield. Conditions to effect the racemization of the undesired (+)-PZQamine are now being sought.[68]

At the same time as this procedure was being discovered by an open approach, another contract research organisation was asked (by WHO/TDR) to look into solutions to the same problem without communication to the open project. This led to the discovery of a complementary resolution (Figure 2B). From an investigation of compounds available in bulk, a resolution of a commercially-available intermediate (**3**) was assayed. Tartaric acid could effect this resolution to provide the enantioenriched intermediate in 37% yield and 94% *ee*. PZQ could be synthesized from enantioenriched **3** by cyclization with chloroacetyl chloride and removal of the benzoyl group, generating (*R*)-PZQamine, which can be taken on to provide enantiopure (*R*)-PZQ as before. A summary of this method was posted to the coordination website when complete.[50]

Full experimental details for the open process may be found in this paper. Readers are encouraged to review, evaluate and contribute to refining the resolutions online by addressing current weaknesses (e.g., the need for a chlorinated solvent extraction process in the initial PZQ hydrolysis). Both processes show sufficient promise in terms of cost on a lab scale (simple methodology, inexpensive resolving agents, good yields and efficiencies) that costs approaching those needed should be attainable upon scale-up; the processes are therefore being examined by WHO/TDR on a kilogram scale for economic viability. The routes found are quite similar. An advantage of the approach discovered by the CRO is its use of tartaric acid itself, as opposed to a derivative, but the derivatization employed in the open approach is straightforward. Which route is adopted depends to some extent on the method(s) currently employed in the commercial manufacture of the API, and perhaps surprisingly this information is not readily available. The ton-scale availability of **3** implies its use in the synthesis of PZQ, presumably *via* the original Merck process,[7] yet to the best of our knowledge the CRO manufacturing PZQ for the Schistosomiasis Control Initiative (Shin Poong, South Korea) were employing a different approach[39] that generated PZQamine **2** as an intermediate, implying a similar availability of that material in quantity. The open source approach is the basis of an educational project in which students from around the world are encouraged to collaborate in further optimization. (Interested students and laboratory instructors can view the experiments and collaborate on the relevant website[69]).

It is clear that the availability of a new synthetic route, even if it is economically viable, does not translate automatically into a product available to the end-user. Additional elements must be taken into consideration including the regulatory requirements for further studies (chemistry, manufacturing & control; non-clinical; and clinical). While the time and costs associated with this process are expected to be significantly less than with a typical new chemical entity, they are yet to be quantified and supported. WHO/TDR is actively seeking commercial partners potentially interested in pursuing this project as well as sources of funding.

## Supporting Information

Figure S1 HPLC trace for (*rac*)-PZQ.
(JPG)

Figure S2 HPLC trace for (*rac*)-PZQamine.
(JPG)

Figure S3 ^1^H NMR spectrum for (*rac*)-PZQamine.
(PDF)

Figure S4 ^13^C NMR spectrum for (*rac*)-PZQamine.
(PDF)

Figure S5 Plot of optical purity *vs.* optical rotation for PZQ.
(TIF)

Figure S6 Plot of optical purity *vs.* optical rotation for PZQamine.
(TIF)

Figure S7 ^1^H NMR spectrum of (–)-dibenzoyl-L-tartaric acid.
(PDF)

Figure S8 ^13^C NMR spectrum of (–)-dibenzoyl-L-tartaric acid.
(PDF)

Figure S9 ^1^H NMR spectrum of (+)-Di-*p*-anisoyl-D-tartaric acid.
(PDF)

Figure S10 ^13^C NMR spectrum of (+)-Di-*p*-anisoyl-D-tartaric acid.
(PDF)

Figure S11 HPLC trace for *R*-(–)-PZQ.
(JPG)

Table S1 Relationship between optical purity and optical rotation for PZQ.
(DOCX)

Table S2 Relationship between optical purity and optical rotation for PZQamine.
(DOCX)

Text S1 Evaluation of HPLC methods for analysis of (*rac*)-PZQamine.
(PDF)

Text S2 Contributors to The Synaptic Leap project.
(DOCX)
