# Supplementary figures and images for: Resolution of Praziquantel
Source: PLoS Negl Trop Dis. 2011 Sep 20;5(9):e1260. doi: 10.1371/journal.pntd.0001260 (PMC3176743; doi:10.1371/journal.pntd.0001260)

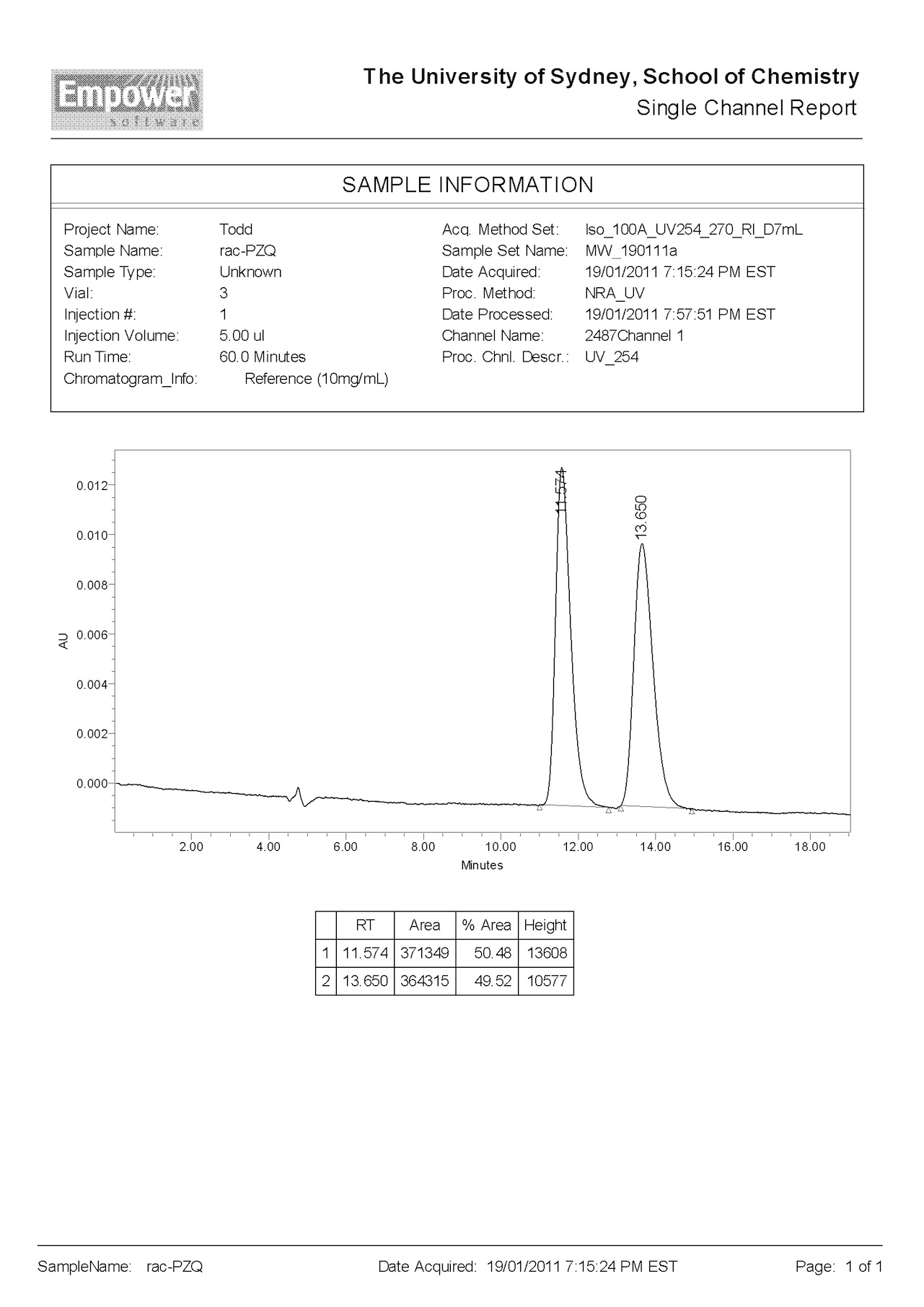

Supplement: Figure S1 — HPLC trace for (rac)-PZQ. (JPG) [file pntd.0001260.s001.jpg]

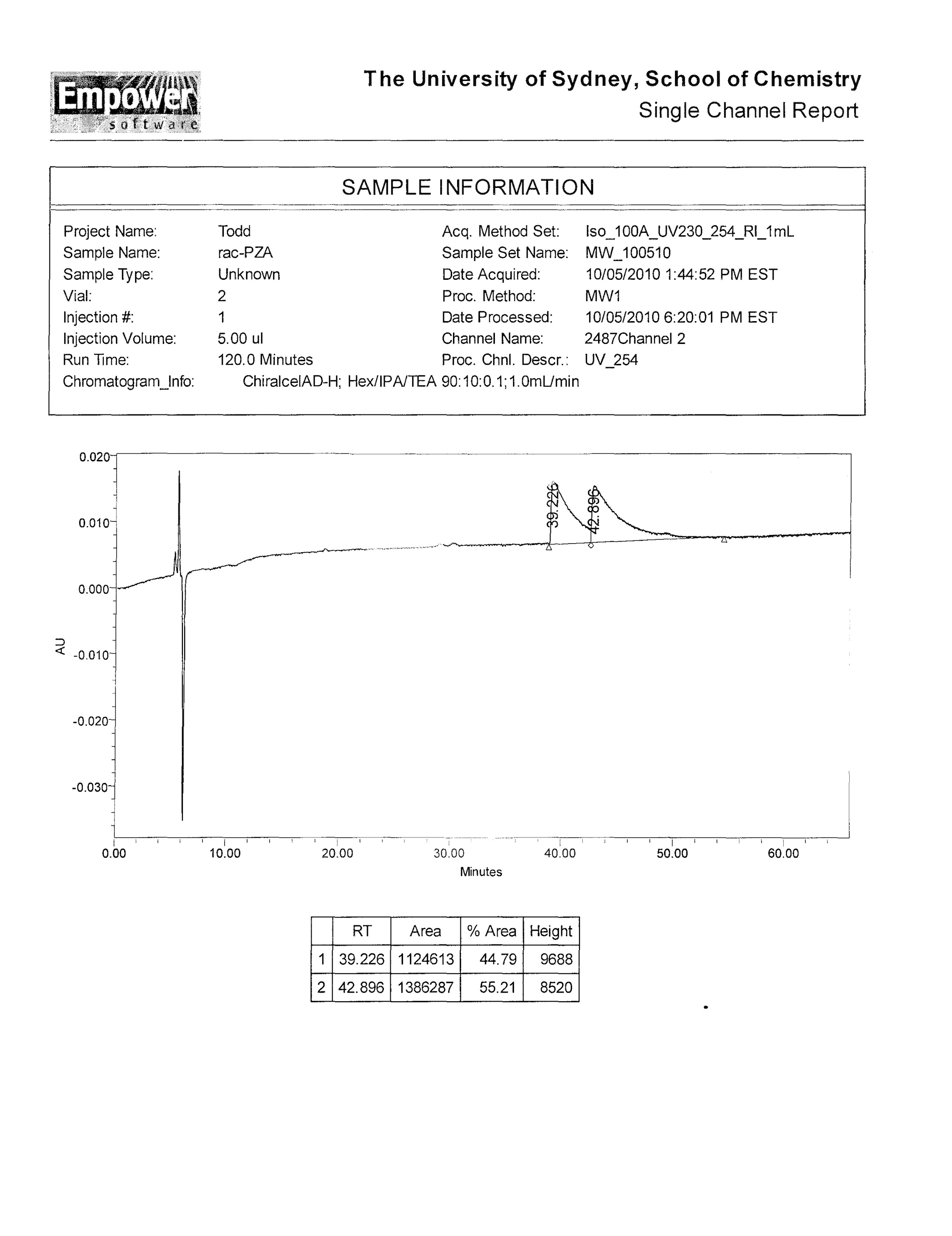

Supplement: Figure S2 — HPLC trace for (rac)-PZQamine. (JPG) [file pntd.0001260.s002.jpg]

# Praziquanamin

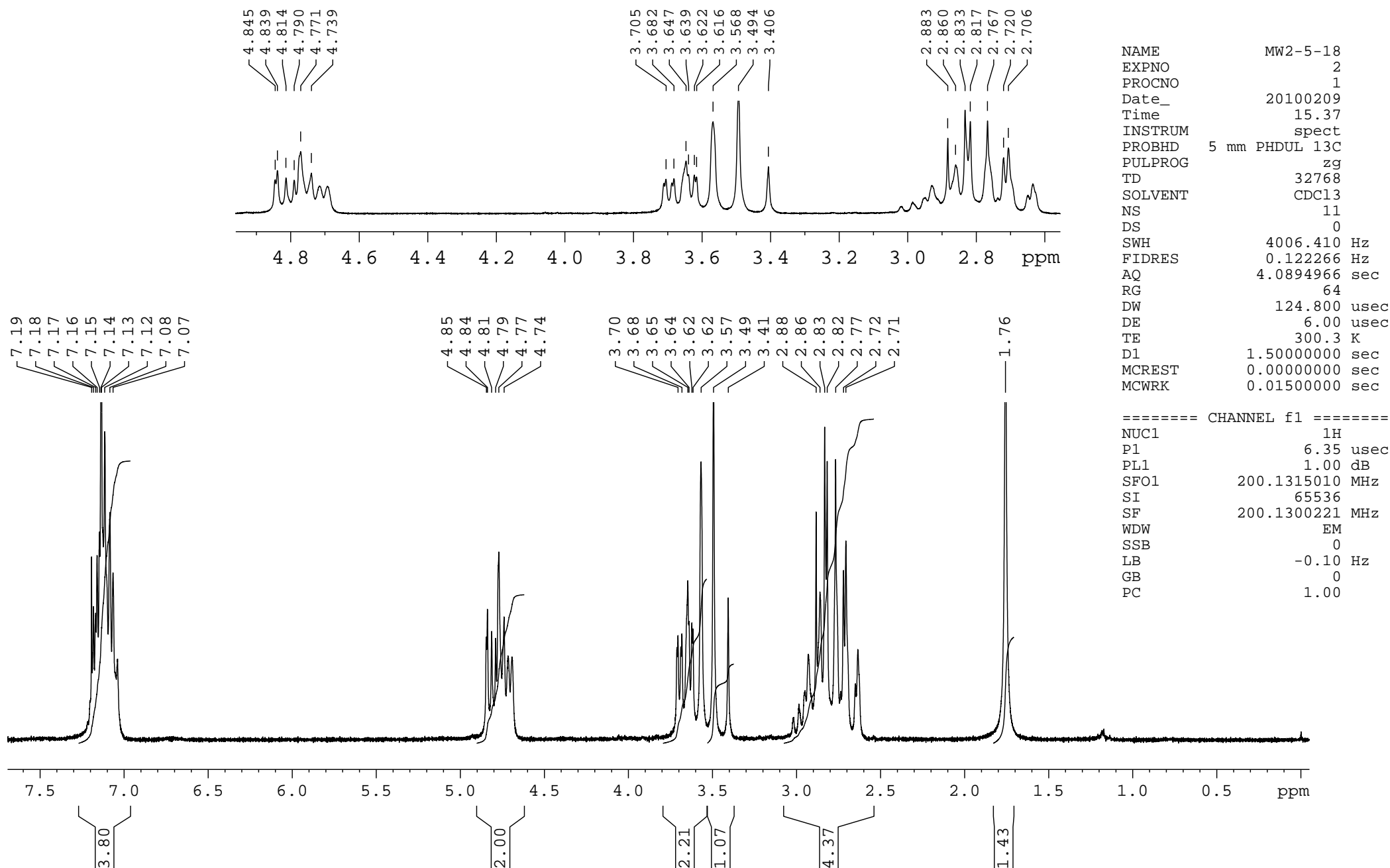

Supplement: Figure S3 — 1H NMR spectrum for (rac)-PZQamine. (PDF) [file pntd.0001260.s003.pdf]

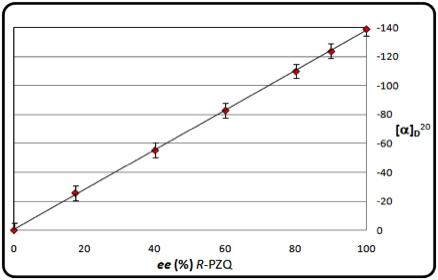

Supplement: Figure S5 — Plot of optical purity vs. optical rotation for PZQ. (TIF) [file pntd.0001260.s005.tif]

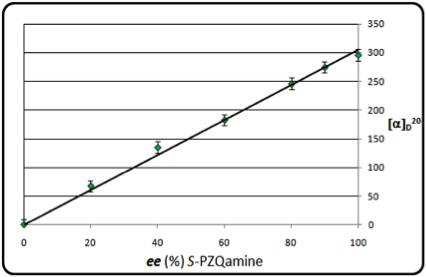

Supplement: Figure S6 — Plot of optical purity vs. optical rotation for PZQamine. (TIF) [file pntd.0001260.s006.tif]

MW10-6 (+)-dibenzoyl-D-tartaric acid after recrystallization from i-PrOH/hexane

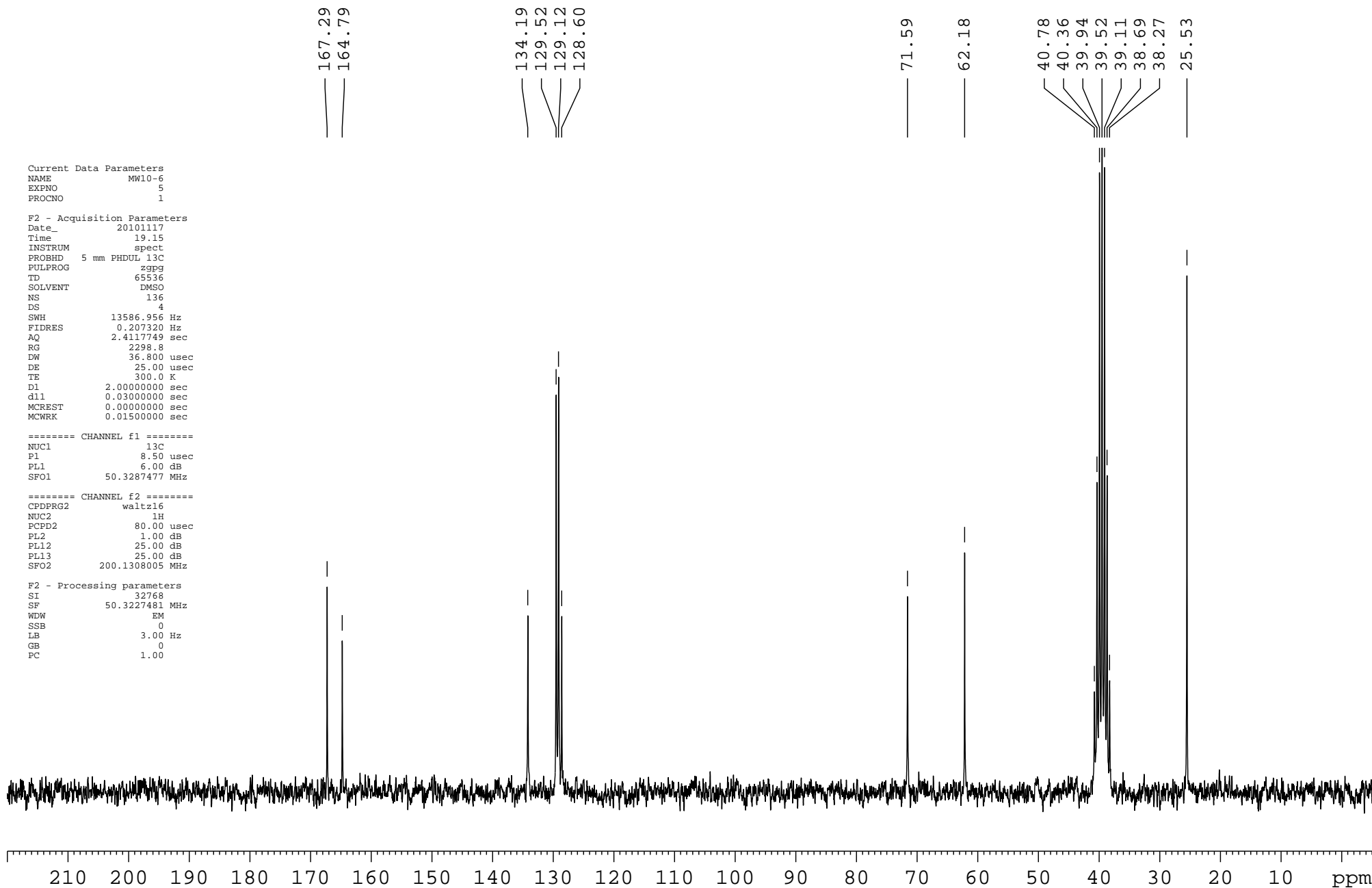

Supplement: Figure S8 — 13C NMR spectrum of (–)-dibenzoyl-L-tartaric acid. (PDF) [file pntd.0001260.s008.pdf]

MW46-2 (+)-p-dianisoly-D-tartaric acid in DMSO (4. precipitation from water)

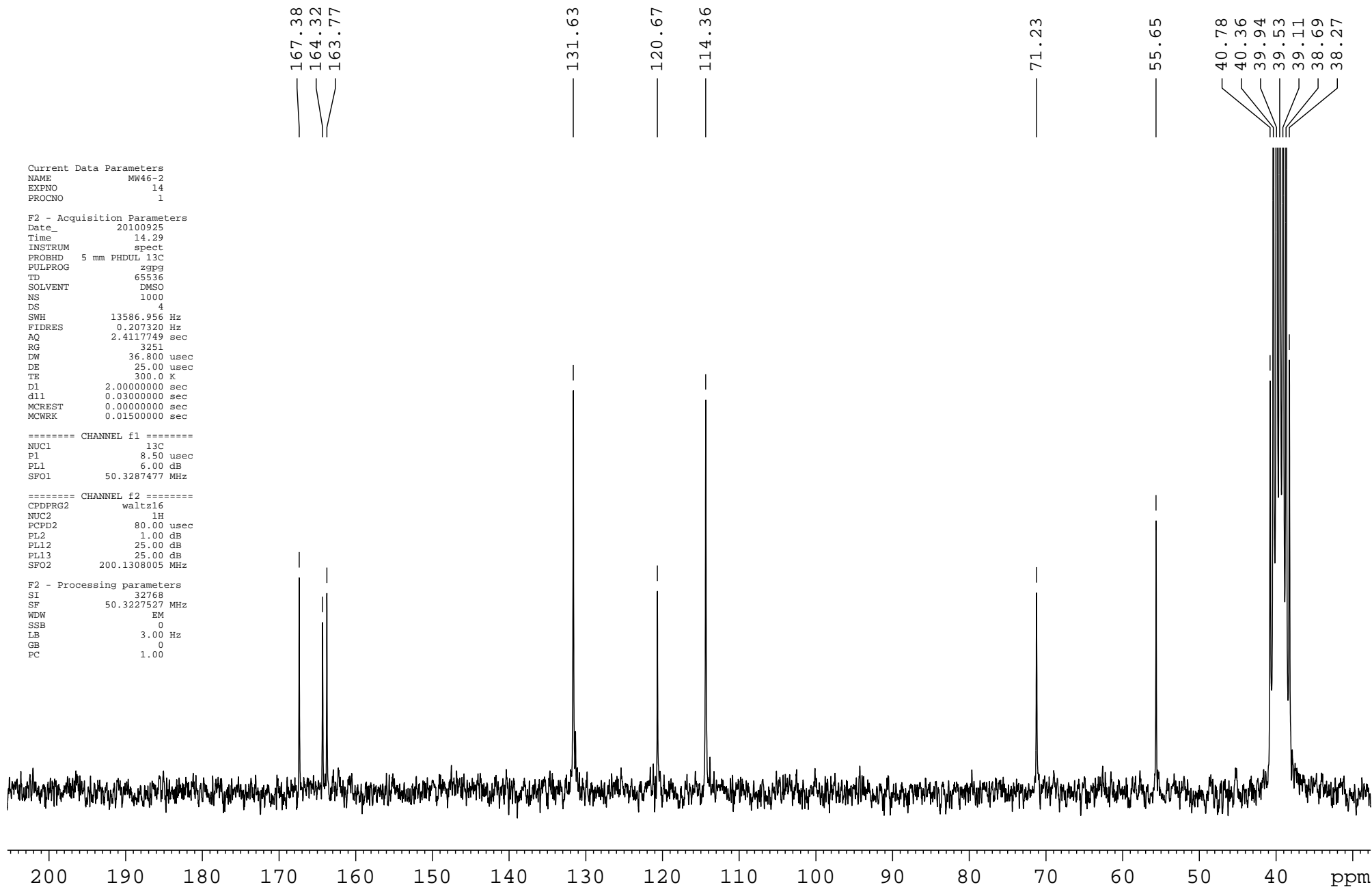

Supplement: Figure S10 — 13C NMR spectrum of (+)-Di-p-anisoyl-D-tartaric acid. (PDF) [file pntd.0001260.s010.pdf]

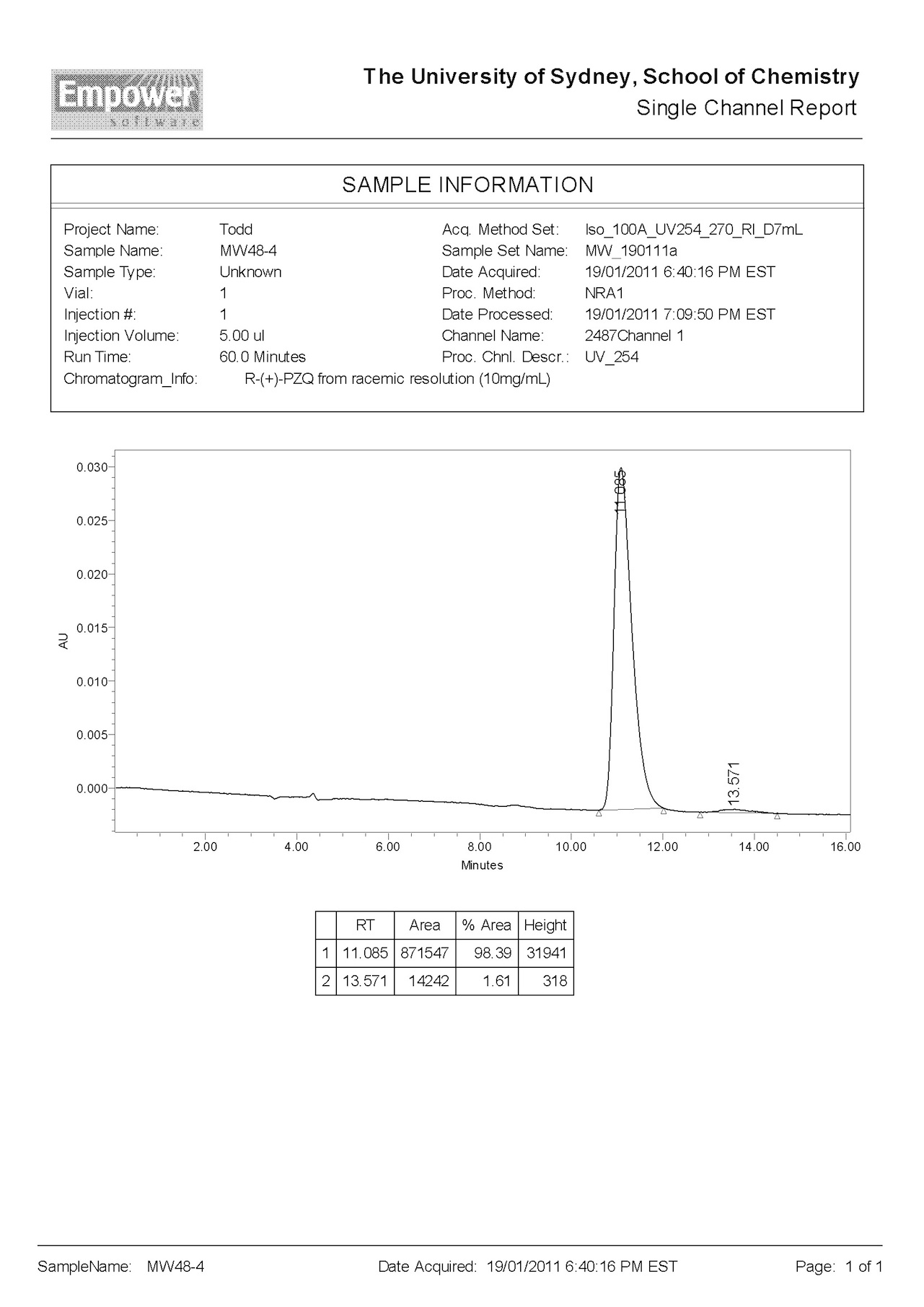

Supplement: Figure S11 — HPLC trace for R-(–)-PZQ. (JPG) [file pntd.0001260.s011.jpg]
