# Supplementary material for: Resolution of Praziquantel
Source: PLoS Negl Trop Dis. 2011 Sep 20;5(9):e1260. doi: 10.1371/journal.pntd.0001260 (PMC3176743; doi:10.1371/journal.pntd.0001260)
Supplement: Figure S4 — 13C NMR spectrum for (rac)-PZQamine. (PDF) [file pntd.0001260.s004.pdf]

Praziquanamin

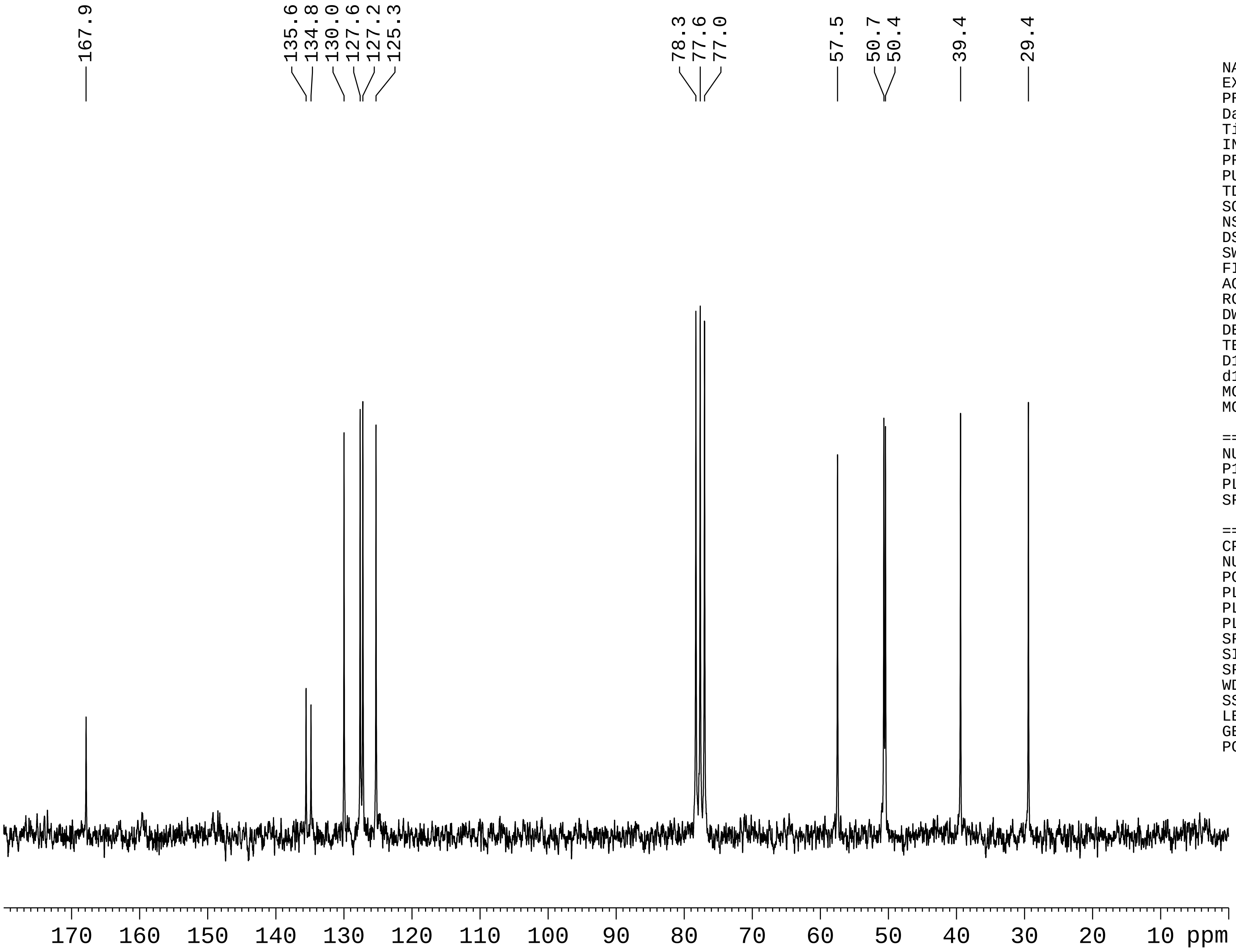

NAME	MW2-5-18	
EXPNO	3	
PROCNO	1	
Date_	20100209	
Time	22.52	
INSTRUM	spect	
PROBHD	5 mm PHDUL 13C	
PULPROG	zgpg	
TD	65536	
SOLVENT	CDCl3	
NS	392	
DS	4	
SWH	13586.956	Hz
FIDRES	0.207320	Hz
AQ	2.4117749	sec
RG	1824.55	
DW	36.800	usec
DE	25.00	usec
TE	300.4	K
D1	2.00000000	sec
d11	0.03000000	sec
MCREST	0.00000000	sec
MCWRK	0.01500000	sec

======== CHANNEL f1 ========

NUC1	13C	
P1	8.50	usec
PL1	6.00	dB
SFO1	50.3287477	MHz

======== CHANNEL f2 ========

CPDPRG2	waltz16	
NUC2	1H	
PCPD2	80.00	usec
PL2	1.00	dB
PL12	25.00	dB
PL13	25.00	dB
SFO2	200.1308005	MHz
SI	65536	
SF	50.3227006	MHz
WDW	EM	
SSB	0	
LB	3.00	Hz
GB	0	
PC	1.00	
