# Supplementary material for: Resolution of Praziquantel
Source: PLoS Negl Trop Dis. 2011 Sep 20;5(9):e1260. doi: 10.1371/journal.pntd.0001260 (PMC3176743; doi:10.1371/journal.pntd.0001260)

MW46-6 4. heating with toluene

Current Data Parameters
NAME MW46-6
EXPNO 6
PROCNO 1

F2 - Acquisition Parameters
Date_ 20101203
Time 12.21
INSTRUM spect
PROBHD 5 mm PHDUL 13C
PULPROG zg
TD 32768
SOLVENT DMSO
NS 19
DS 0
SWH 4006.410 Hz
FIDRES 0.122266 Hz
AQ 4.0894966 sec
RG 90.5
DW 124.800 usec
DE 6.00 usec
TE 300.0 K
D1 1.50000000 sec
MCREST 0.00000000 sec
MCWRK 0.01500000 sec

======== CHANNEL f1 ========
NUC1 1H
P1 6.35 usec
PL1 1.00 dB
SFO1 200.1315010 MHz

F2 - Processing parameters
SI 16384
SF 200.1300052 MHz
WDW no
SSB 0
LB 0.00 Hz
GB 0
PC 1.00

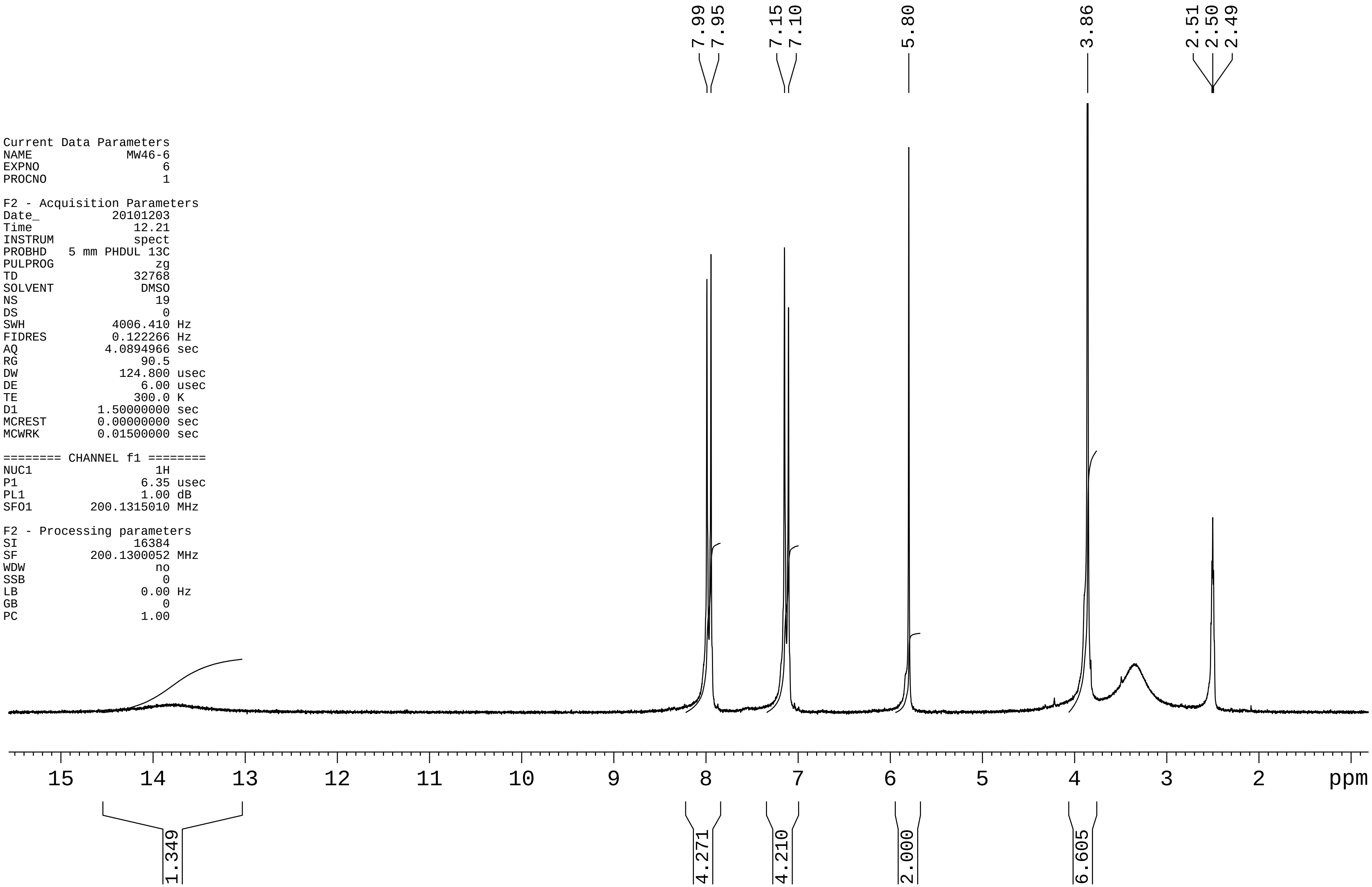

Supplement: Figure S9 — 1H NMR spectrum of (+)-Di-p-anisoyl-D-tartaric acid. (PDF) [file pntd.0001260.s009.pdf]
