# Supplementary material for: Resolution of Praziquantel
Source: PLoS Negl Trop Dis. 2011 Sep 20;5(9):e1260. doi: 10.1371/journal.pntd.0001260 (PMC3176743; doi:10.1371/journal.pntd.0001260)
Supplement: Table S1 — Relationship between optical purity and optical rotation for PZQ. (DOCX) [file pntd.0001260.s012.docx]

| **Volume (*R*)-(–)-PZQ (mL)** | **Volume (*S*)-(+)-PZQ (mL)** | ***ee*** | **[α]** |
| --- | --- | --- | --- |
| 1 | 0 | 100 | -139 |
| 0.95 | 0.05 | 90 | -123.7 |
| 0.9 | 0.1 | 80 | -109.9 |
| 0.8 | 0.2 | 60 | -82.9 |
| 0.7 | 0.3 | 40 | -55.3 |
| 0.587 | 0.413 | 17.4 | -25.8 |
| 0.5 | 0.5 | 0 | -0.1 |

Table S1. Relationship between optical rotation and optical purity for PZQ
