# Supplementary material for: Resolution of Praziquantel
Source: PLoS Negl Trop Dis. 2011 Sep 20;5(9):e1260. doi: 10.1371/journal.pntd.0001260 (PMC3176743; doi:10.1371/journal.pntd.0001260)
Supplement: Table S2 — Relationship between optical purity and optical rotation for PZQamine. (DOCX) [file pntd.0001260.s013.docx]

| **Volume (*S*)-(+)-PZQamine (mL)** | **Volume *rac*-PZQamine solution** | ***ee*** | **[α]** |
| --- | --- | --- | --- |
| 1 | 0 | 100 | 296 |
| 0.9 | 0.1 | 90 | 275 |
| 0.8 | 0.2 | 80 | 246 |
| 0.6 | 0.4 | 60 | 183 |
| 0.4 | 0.6 | 40 | 135 |
| 0.2 | 0.8 | 20 | 67.7 |
| 0 | 1 | 0 | 0 |

Table S2. Relationship between optical rotation and optical purity for PZQamine
