# Supplementary material for: Resolution of Praziquantel
Source: PLoS Negl Trop Dis. 2011 Sep 20;5(9):e1260. doi: 10.1371/journal.pntd.0001260 (PMC3176743; doi:10.1371/journal.pntd.0001260)
Supplement: Text S1 — Evaluation of HPLC methods for analysis of (rac)-PZQamine. (PDF) [file pntd.0001260.s014.pdf]

SYNCOM
Designing chemistry

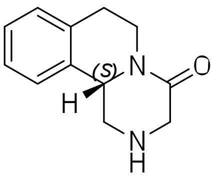

(S)-(+)

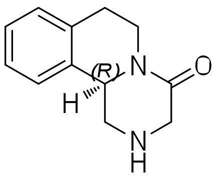

(R)-(-)

Chiral HPLC separation praziquanamine enantiomers

Data File D:\DATA\SE18MAY\18051000.D Sample Name: JSN147927-RAC

```
=====================================================================
Injection Date  : 5/18/2010 9:55:50 AM          Seq. Line :   1
Sample Name     : JSN147927-RAC                  Location : Vial 21
Acq. Operator   : JGR                                 Inj :   1
                                               Inj Volume : 2 µl

Method          : C:\HPCHEM\1\METHODS\19671.M
Last changed    : 5/18/2010 9:20:51 AM by EEN
Chiralcel OJ-H
Heptane/EtOH/Et2NH (60/40/0.2)
0.5 ml/min
```

racemate OJ-H

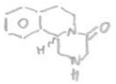

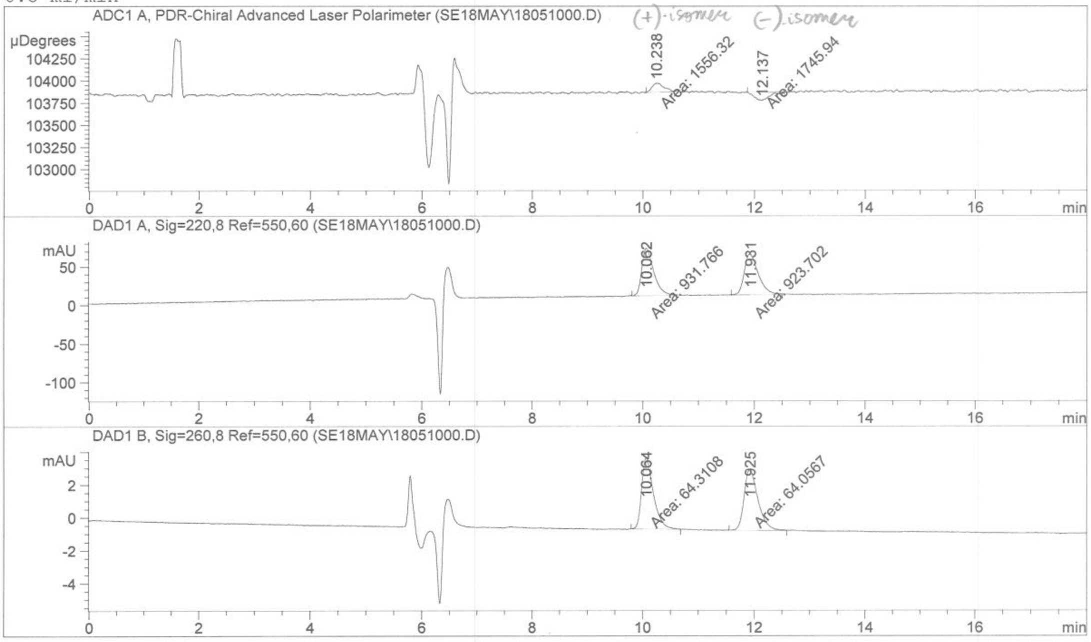


```
=====================================================================
                         Area Percent Report
=====================================================================

Sorted By             :      Signal
Multiplier            :      1.0000
Dilution              :      1.0000
Use Multiplier & Dilution Factor with ISTDs


Signal 1: ADC1 A, PDR-Chiral Advanced Laser Polarimeter
```

Peak #	RetTime [min]	Type	Width [min]	Area µDegrees*	Height µDegrees	Area %
1	10.238	MM	0.2503	1556.32312	103.63797	47.1290
2	12.137	PM N	0.3164	1745.94006	91.97827	52.8710
Totals :				3302.26318	195.61624	

Results obtained with enhanced integrator!

VISITING ADDRESS POSTAL ADDRESS TELEPHONE FAX E-MAIL TRADE REGISTER INTERNET
Kadijk 3, 9747 AT Groningen, The Netherlands P.O. Box 2253, 9704 CE Groningen +31(0)505757272 +31(0)505757399 info@syncom.nl 020 411 05 www.syncom.nl

SYNCOM
Designing chemistry

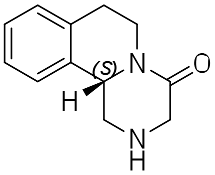

(S)-(+)

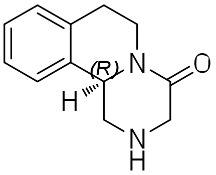

(R)-(-)

Chiral HPLC separation praziquanamine enantiomers

Data File D:\DATA\SE18MAY\18051000.D

Sample Name: JSN147927-RAC

Signal 2: DAD1 A, Sig=220,8 Ref=550,60

Peak #	RetTime [min]	Type	Width [min]	Area [mAU*s]	Height [mAU]	Area %
1	10.062	MM	0.2517	931.76593	61.70352	50.2173
2	11.931	MM	0.2905	923.70172	52.98610	49.7827
Totals :				1855.46765	114.68961	

Results obtained with enhanced integrator!

Signal 3: DAD1 B, Sig=260,8 Ref=550,60

Peak #	RetTime [min]	Type	Width [min]	Area [mAU*s]	Height [mAU]	Area %
1	10.064	MM	0.2476	64.31081	4.32948	50.0990
2	11.925	MM	0.2866	64.05672	3.72544	49.9010
Totals :				128.36753	8.05493	

Results obtained with enhanced integrator!

===

*** End of Report ***

VISITING ADDRESS POSTAL ADDRESS TELEPHONE FAX E-MAIL TRADE REGISTER INTERNET

Kadijk 3, 9747 AT Groningen, The Netherlands P.O. Box 2253, 9704 CE Groningen +31(0)505757272 +31(0)505757399 info@syncom.nl 020 411 05 www.syncom.nl

SYNCOM
Designing chemistry

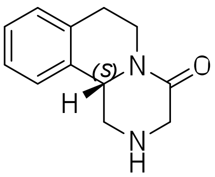

(S)-(+)

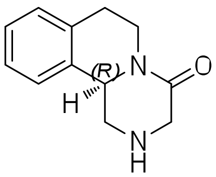

(R)-(-)

Chiral HPLC separation praziquanamine enantiomers

Data File D:\DATA\SEQ17MAY\17051008.D   Sample Name: JSN147927-RAC

===

```
Injection Date  : 5/17/2010 7:48:00 PM            Seq. Line :    4
Sample Name     : JSN147927-RAC                    Location : Vial 21
Acq. Operator   : JGR                                   Inj :    1
                                                 Inj Volume : 2 µl

Method          : C:\HPCHEM\1\METHODS\C1ETOH-B.M
Last changed    : 4/29/2010 2:05:15 PM by jln
Chiralpak IA
Heptane/EtOH/Et2NH (60/40/0.2)
0.7 ml/min
```

IA

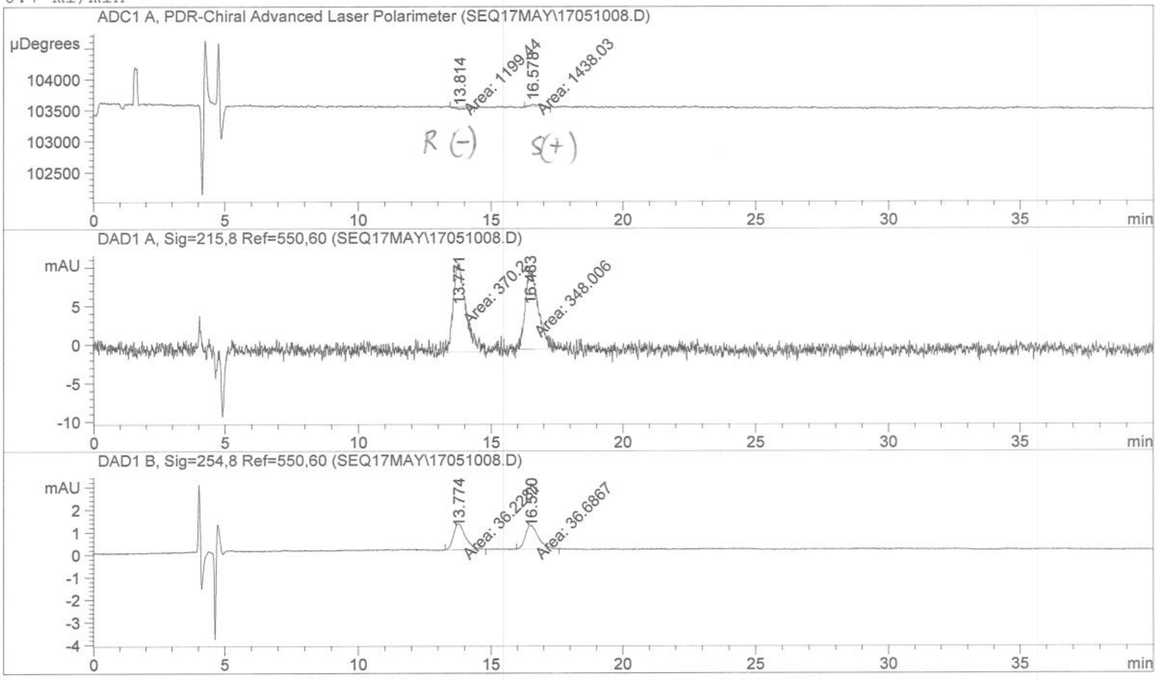

===
Area Percent Report
===

```
Sorted By                 :      Signal
Multiplier                :      1.0000
Dilution                  :      1.0000
Use Multiplier & Dilution Factor with ISTDs
```

Signal 1: ADC1 A, PDR-Chiral Advanced Laser Polarimeter

Peak #	RetTime [min]	Type	Width [min]	Area µDegrees*	Height µDegrees	Area %
1	13.814	MP N	0.4440	1199.43567	45.02583	45.4769
2	16.578	MM	0.4977	1438.02808	48.15579	54.5231

Totals : 2637.46375   93.18163

Results obtained with enhanced integrator!

VISITING ADDRESS POSTAL ADDRESS TELEPHONE FAX E-MAIL TRADE REGISTER INTERNET

Kadijk 3, 9747 AT Groningen, The Netherlands P.O. Box 2253, 9704 CE Groningen +31(0)505757272 +31(0)505757399 info@syncom.nl 020 411 05 www.syncom.nl

Chiral HPLC separation praziquanamine enantiomers

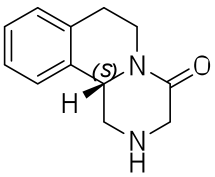

(S)-(+)

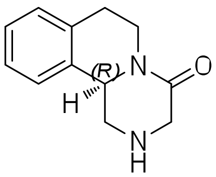

(R)-(-)

Data File D:\DATA\SEQ17MAY\17051008.D

Sample Name: JSN147927-RA

Signal 2: DAD1 A, Sig=215,8 Ref=550,60

Peak #	RetTime [min]	Type	Width [min]	Area [mAU*s]	Height [mAU]	Area %
1	13.771	MM	0.5362	370.19980	11.50707	51.5451
2	16.483	MM	0.5433	348.00555	10.67497	48.4549
Totals :				718.20535	22.18204	

Results obtained with enhanced integrator!

Signal 3: DAD1 B, Sig=254,8 Ref=550,60

Peak #	RetTime [min]	Type	Width [min]	Area [mAU*s]	Height [mAU]	Area %
1	13.774	MM	0.5226	36.22811	1.15529	49.6855
2	16.520	MM	0.5733	36.68669	1.06650	50.3145
Totals :				72.91480	2.22179	

Results obtained with enhanced integrator!

*** End of Report ***

VISITING ADDRESS POSTAL ADDRESS TELEPHONE FAX E-MAIL TRADE REGISTER INTERNET

Kadijk 3, 9747 AT Groningen, The Netherlands P.O. Box 2253, 9704 CE Groningen +31(0)505757272 +31(0)505757399 info@syncom.nl 020 411 05 www.syncom.nl

SYNCOM
Designing chemistry

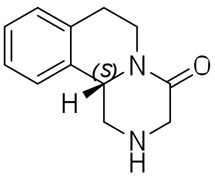

(S)-(+)

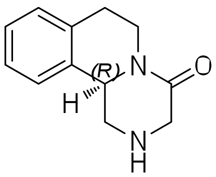

(R)-(-)

Chiral HPLC separation praziquanamine enantiomers

Data File D:\DATA\SEQ17MAY\17051009.D Sample Name: JSN147927-RAC

===

```
Injection Date   : 5/17/2010 8:50:31 PM           Seq. Line :    5
Sample Name      : JSN147927-RAC                   Location : Vial 21
Acq. Operator    : JGR                                  Inj :    1
                                                 Inj Volume : 4 µl

Method           : C:\HPCHEM\1\METHODS\C2ETOH-B.M
Last changed     : 4/29/2010 4:37:34 PM by kbr
Chiralpak AS-H
Heptane/EtOH/Et2NH (60/40/0.2)
0.5 ml/mi
```

AS-H

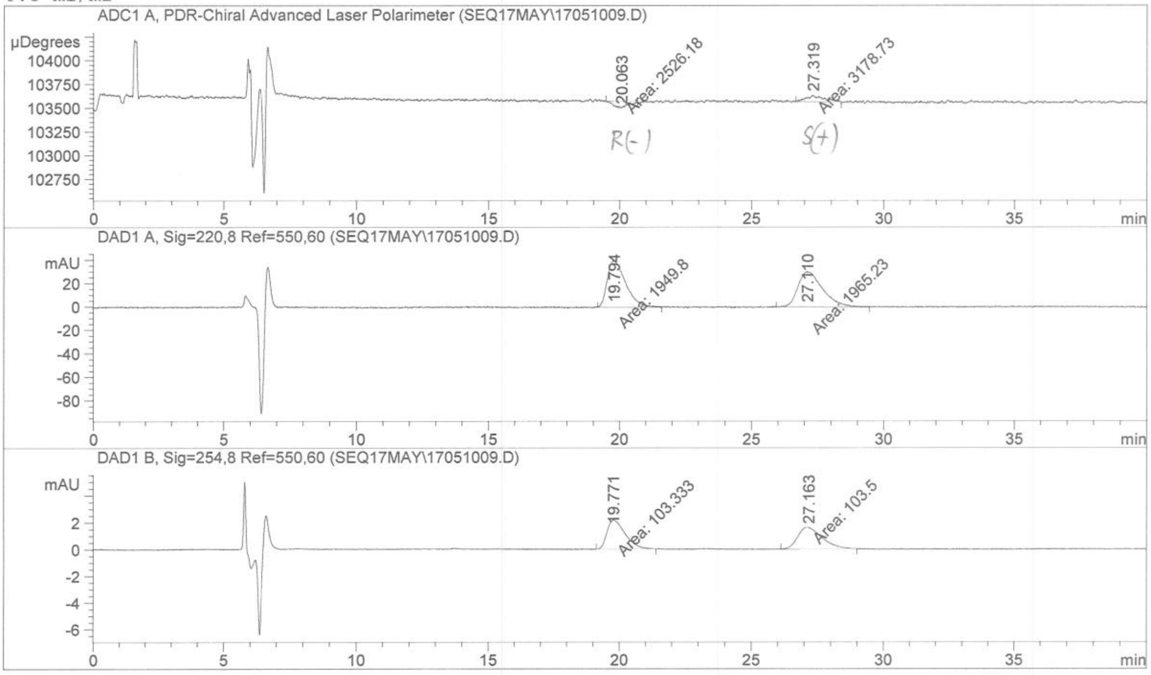

===

Area Percent Report

===

```
Sorted By            :      Signal
Multiplier           :      1.0000
Dilution             :      1.0000
Use Multiplier & Dilution Factor with ISTDs
```

Signal 1: ADC1 A, PDR-Chiral Advanced Laser Polarimeter

Peak #	RetTime [min]	Type	Width [min]	Area µDegrees*	Height µDegrees	Area %
1	20.063	PM N	0.6120	2526.17847	68.79725	44.2808
2	27.319	MM	0.7871	3178.73193	67.30531	55.7192
Totals :				5704.91040	136.10255	

Results obtained with enhanced integrator!

VISITING ADDRESS POSTAL ADDRESS TELEPHONE FAX E-MAIL TRADE REGISTER INTERNET
Kadijk 3, 9747 AT Groningen, The Netherlands P.O. Box 2253, 9704 CE Groningen +31(0)505757272 +31(0)505757399 info@syncom.nl 020 411 05 www.syncom.nl

SYNCOM

Designing chemistry

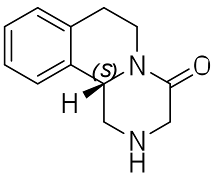

(S)-(+)

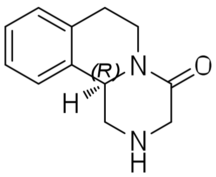

(R)-(-)

Chiral HPLC separation praziquanamine enantiomers

Data File D:\DATA\SEQ17MAY\17051009.D

Sample Name: JSN147927-RAC

Signal 2: DAD1 A, Sig=220,8 Ref=550,60

Peak #	RetTime [min]	Type	Width [min]	Area [mAU*s]	Height [mAU]	Area %
1	19.794	MM	0.8208	1949.80469	39.58933	49.8029
2	27.110	MM	1.0763	1965.23486	30.43194	50.1971

Totals : 3915.03955 70.02127

Results obtained with enhanced integrator!

Signal 3: DAD1 B, Sig=254,8 Ref=550,60

Peak #	RetTime [min]	Type	Width [min]	Area [mAU*s]	Height [mAU]	Area %
1	19.771	MM	0.7978	103.33324	2.15884	49.9597
2	27.163	MM	1.0650	103.49986	1.61965	50.0403

Totals : 206.83309 3.77850

Results obtained with enhanced integrator!

*** End of Report ***

VISITING ADDRESS POSTAL ADDRESS TELEPHONE FAX E-MAIL TRADE REGISTER INTERNET

Kadijk 3, 9747 AT Groningen, The Netherlands P.O. Box 2253, 9704 CE Groningen +31(0)505757272 +31(0)505757399 info@syncom.nl 020 411 05 www.syncom.nl

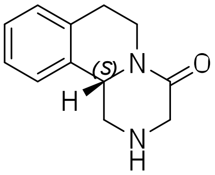

(S)-(+)

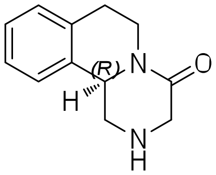

(R)-(-)

Designing chemistry

Chiral HPLC separation praziquanamine enantiomers

Data File D:\DATA\SEQ17MAY\17051012.D   Sample Name: JSN147927-RAC

===

```
Injection Date  : 5/17/2010 11:57:17 PM        Seq. Line :   8
Sample Name     : JSN147927-RAC                 Location : Vial 21
Acq. Operator   : JGR                                Inj :   1
                                              Inj Volume : 2 µl
```

IB

```
Method          : C:\HPCHEM\1\METHODS\C5ETOH-B.M
Last changed    : 4/29/2010 4:38:21 PM by kbr
Chiralpak IB
Heptane/EtOH/Et2NH (60/40/0.2)
0.7 ml/min
```

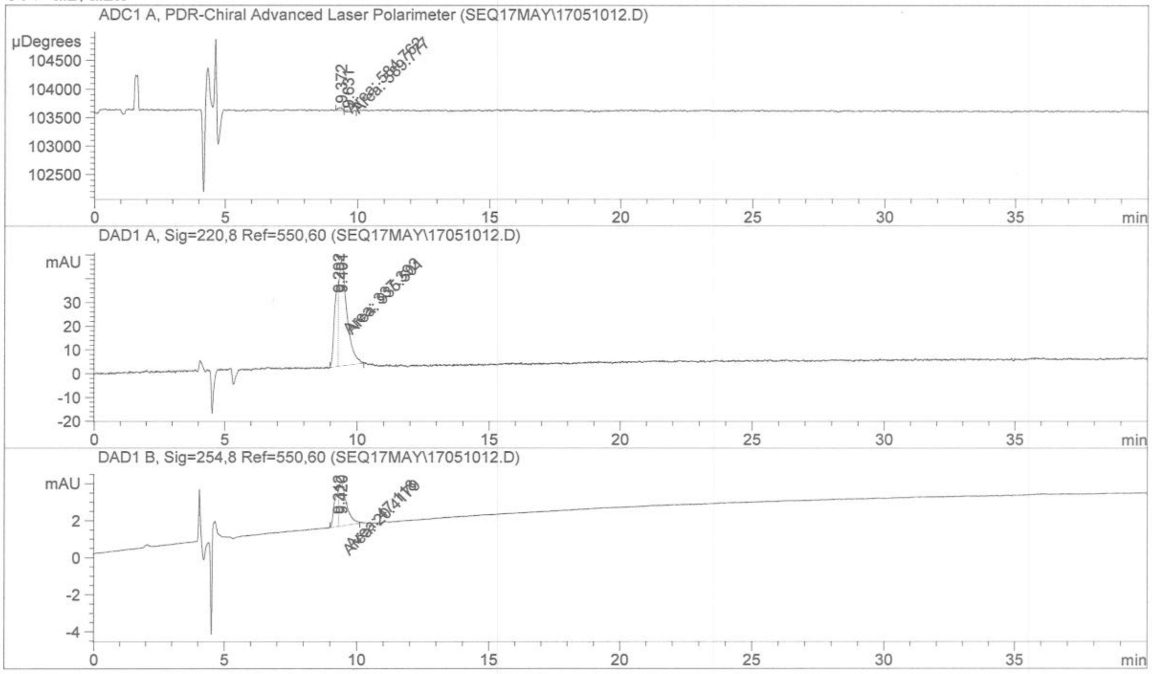

===

Area Percent Report

===

```
Sorted By             :      Signal
Multiplier            :      1.0000
Dilution              :      1.0000
Use Multiplier & Dilution Factor with ISTDs
```

Signal 1: ADC1 A, PDR-Chiral Advanced Laser Polarimeter

Peak #	RetTime [min]	Type	Width [min]	Area µDegrees*	Height µDegrees	Area %
1	9.372	MM	0.1877	584.76245	51.92204	49.7865
2	9.631	MM N	0.2665	589.77692	36.87745	50.2135
Totals :				1174.53937	88.79948	

Results obtained with enhanced integrator!

VISITING ADDRESS | POSTAL ADDRESS | TELEPHONE | FAX | E-MAIL | TRADE REGISTER | INTERNET

Kadijk 3, 9747 AT Groningen, The Netherlands | P.O. Box 2253, 9704 CE Groningen | +31(0)505757272 | +31(0)505757399 | info@syncom.nl | 020 411 05 | www.syncom.nl

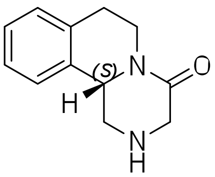

(S)-(+)

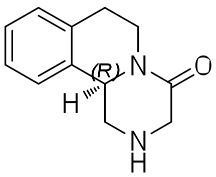

(R)-(-)

Chiral HPLC separation praziquanamine enantiomers

Data File D:\DATA\SEQ17MAY\17051012.D

Sample Name: JSN147927-RAC

Signal 2: DAD1 A, Sig=220,8 Ref=550,60

Peak #	RetTime [min]	Type	Width [min]	Area [mAU*s]	Height [mAU]	Area %
1	9.292	MF	0.1668	337.39240	33.72030	26.5059
2	9.401	FM	0.3499	935.50116	44.55696	73.4941

Totals : 1272.89355 78.27726

Results obtained with enhanced integrator!

Signal 3: DAD1 B, Sig=254,8 Ref=550,60

Peak #	RetTime [min]	Type	Width [min]	Area [mAU*s]	Height [mAU]	Area %
1	9.312	MF	0.1775	20.41792	1.91735	30.2327
2	9.420	FM	0.2385	47.11800	2.39324	69.7673

Totals : 67.53593 4.31060

Results obtained with enhanced integrator!

===

*** End of Report ***

VISITING ADDRESS POSTAL ADDRESS TELEPHONE FAX E-MAIL TRADE REGISTER INTERNET

Kadijk 3, 9747 AT Groningen, The Netherlands P.O. Box 2253, 9704 CE Groningen +31(0)505757272 +31(0)505757399 info@syncom.nl 020 411 05 www.syncom.nl

Designing chemistry

Chiral HPLC separation praziquanamine enantiomers

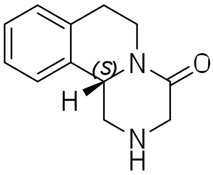

(S)-(+)

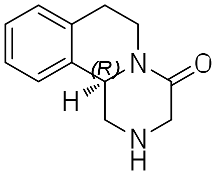

(R)-(-)

Data File D:\DATA\SEQ17MAY\17051010.D   Sample Name: JSN147927-RAC

===

```
Injection Date  : 5/17/2010 9:52:48 PM          Seq. Line :    6
Sample Name     : JSN147927-RAC                  Location : Vial 21
Acq. Operator   : JGR                                 Inj :    1
                                               Inj Volume : 2 µl

Acq. Method     : C:\HPCHEM\1\METHODS\C3ETOH-B.M
Last changed    : 5/17/2010 9:52:39 PM by JGR
                  (modified after loading)
Analysis Method : C:\HPCHEM\1\METHODS\C3ETOH-B.M
Last changed    : 4/29/2010 4:37:51 PM by kbr
Chiralpak OD-H
Heptane/EtOH/Et2NH (60/40/0.2)
0.5 ml/min
```

OD-H

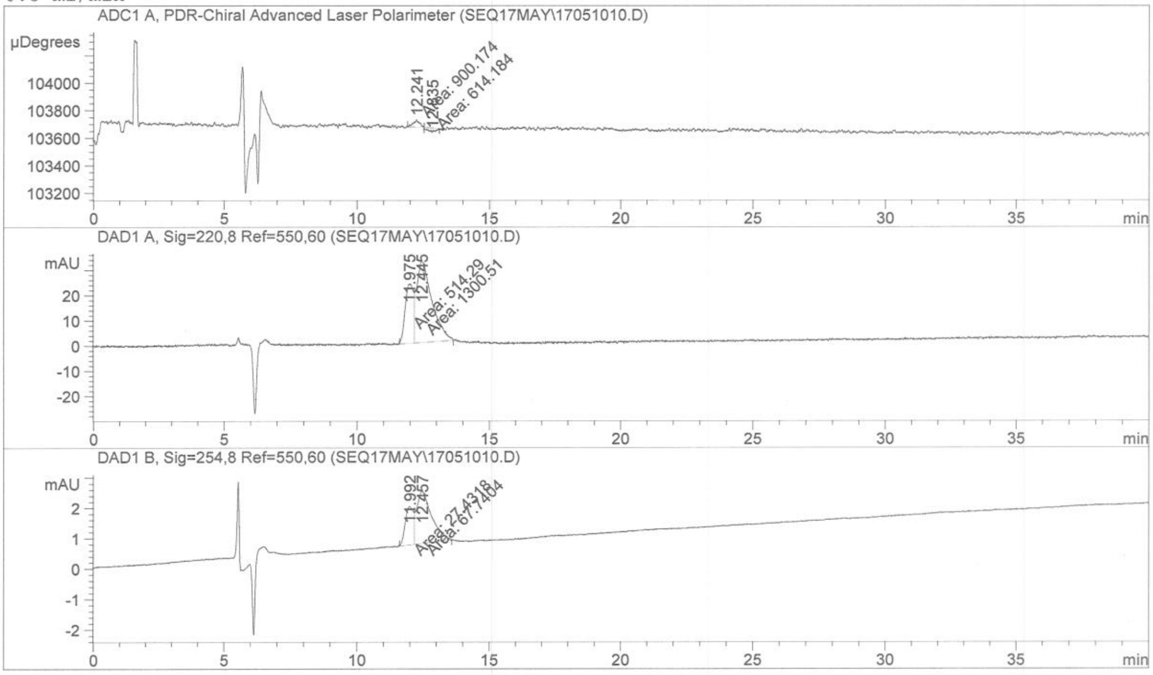

===

Area Percent Report

===

Sorted By : Signal
Multiplier : 1.0000
Dilution : 1.0000
Use Multiplier & Dilution Factor with ISTDs

Signal 1: ADC1 A, PDR-Chiral Advanced Laser Polarimeter

Peak #	RetTime [min]	Type	Width [min]	Area µDegrees*	Height µDegrees	Area %
1	12.241	MM	0.2784	900.17419	53.88367	59.4426
2	12.835	MM N	0.3192	614.18396	32.06727	40.5574

Totals : 1514.35815   85.95095

Results obtained with enhanced integrator!

VISITING ADDRESS POSTAL ADDRESS TELEPHONE FAX E-MAIL TRADE REGISTER INTERNET

Kadijk 3, 9747 AT Groningen, The Netherlands P.O. Box 2253, 9704 CE Groningen +31(0)505757272 +31(0)505757399 info@syncom.nl 020 411 05 www.syncom.nl

SYNCOM

Designing chemistry

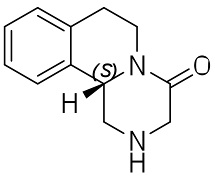

(S)-(+)

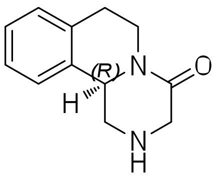

(R)-(-)

Chiral HPLC separation praziquanamine enantiomers

Data File D:\DATA\SEQ17MAY\17051010.D

Sample Name: JSN147927-RAC

Signal 2: DAD1 A, Sig=220,8 Ref=550,60

Peak #	RetTime [min]	Type	Width [min]	Area [mAU*s]	Height [mAU]	Area %
1	11.975	MF	0.3633	514.28986	23.59226	28.3387
2	12.445	FM	0.6720	1300.50854	32.25372	71.6613

Totals : 1814.79840 55.84598

Results obtained with enhanced integrator!

Signal 3: DAD1 B, Sig=254,8 Ref=550,60

Peak #	RetTime [min]	Type	Width [min]	Area [mAU*s]	Height [mAU]	Area %
1	11.992	MF	0.3602	27.43178	1.26920	28.8233
2	12.457	FM	0.6529	67.74045	1.72931	71.1767

Totals : 95.17223 2.99852

Results obtained with enhanced integrator!

*** End of Report ***

VISITING ADDRESS POSTAL ADDRESS TELEPHONE FAX E-MAIL TRADE REGISTER INTERNET

Kadijk 3, 9747 AT Groningen, The Netherlands P.O. Box 2253, 9704 CE Groningen +31(0)505757272 +31(0)505757399 info@syncom.nl 020 411 05 www.syncom.nl

SYNCOM
Designing chemistry

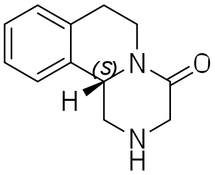

(S)-(+)

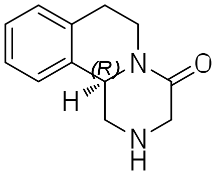

(R)-(-)

Chiral HPLC separation praziquanamine enantiomers

Data File D:\DATA\SEQ17MAY\17051006.D   Sample Name: JSN147927-RAC

===

Injection Date : 5/17/2010 5:53:32 PM   Seq. Line : 2
Sample Name : JSN147927-RAC   Location : Vial 21
Acq. Operator : JGR   Inj : 1
Inj Volume : 4 µl

OJ-H

Method : C:\HPCHEM\1\METHODS\C4ETOH-N.M
Last changed : 5/17/2010 4:32:39 PM by EEN
Chiralcel OJ-H
Heptane/EtOH 60/40
0.5 ml/min

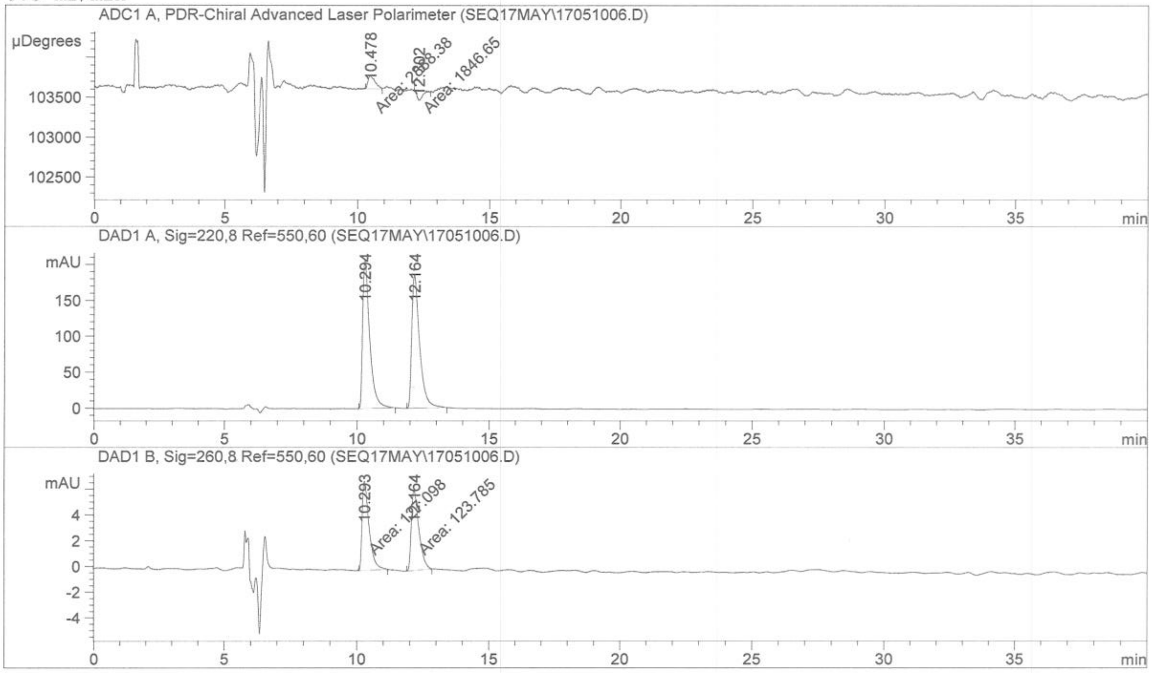

===
Area Percent Report
===

Sorted By : Signal
Multiplier : 1.0000
Dilution : 1.0000
Use Multiplier & Dilution Factor with ISTDs

Signal 1: ADC1 A, PDR-Chiral Advanced Laser Polarimeter

Peak #	RetTime [min]	Type	Width [min]	Area µDegrees*	Height µDegrees	Area %
1	10.478	MM	0.2921	2888.37500	164.81291	61.0002
2	12.302	MM N	0.2472	1846.65137	124.48665	38.9998

Totals : 4735.02637 289.29956

Results obtained with enhanced integrator!

VISITING ADDRESS | POSTAL ADDRESS | TELEPHONE | FAX | E-MAIL | TRADE REGISTER | INTERNET

Kadijk 3, 9747 AT Groningen, The Netherlands | P.O. Box 2253, 9704 CE Groningen | +31(0)505757272 | +31(0)505757399 | info@syncom.nl | 020 411 05 | www.syncom.nl

SYNCOM

Designing chemistry

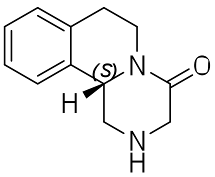

(S)-(+)

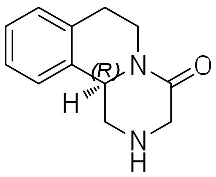

(R)-(-)

Chiral HPLC separation praziquanamine enantiomers

Data File D:\DATA\SEQ17MAY\17051006.D

Sample Name: JSN147927-RAC

Signal 2: DAD1 A, Sig=220,8 Ref=550,60

Peak #	RetTime [min]	Type	Width [min]	Area [mAU*s]	Height [mAU]	Area %
1	10.294	BB	0.2726	3816.03442	206.46095	49.9358
2	12.164	BB	0.3051	3825.85254	185.14583	50.0642
Totals :				7641.88696	391.60678	

Results obtained with enhanced integrator!

Signal 3: DAD1 B, Sig=260,8 Ref=550,60

Peak #	RetTime [min]	Type	Width [min]	Area [mAU*s]	Height [mAU]	Area %
1	10.293	MM	0.3053	127.09833	6.93838	50.6604
2	12.164	MM	0.3319	123.78490	6.21628	49.3396
Totals :				250.88323	13.15466	

Results obtained with enhanced integrator!

==

*** End of Report ***

SYNCOM
Designing chemistry

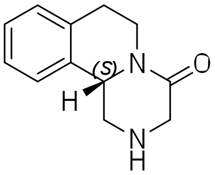

(S)-(+)

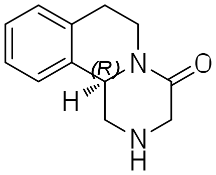

(R)-(-)

Chiral HPLC separation praziquanamine enantiomers

Data File D:\DATA\SEQ17MAY\17051000.D   Sample Name: JSN147927-RAC

```
=====================================================================
Injection Date  : 5/17/2010 2:39:10 PM          Seq. Line :    1
Sample Name     : JSN147927-RAC                  Location : Vial 21
Acq. Operator   : JGR                                 Inj :    1
                                               Inj Volume : 4 µl
Method          : C:\HPCHEM\1\METHODS\C1ETOH-N.M
Last changed    : 5/17/2010 2:29:33 PM by JGR
Chiralpak IA
Heptane/EtOH 80/20
0.7 ML/MIN
```

IA

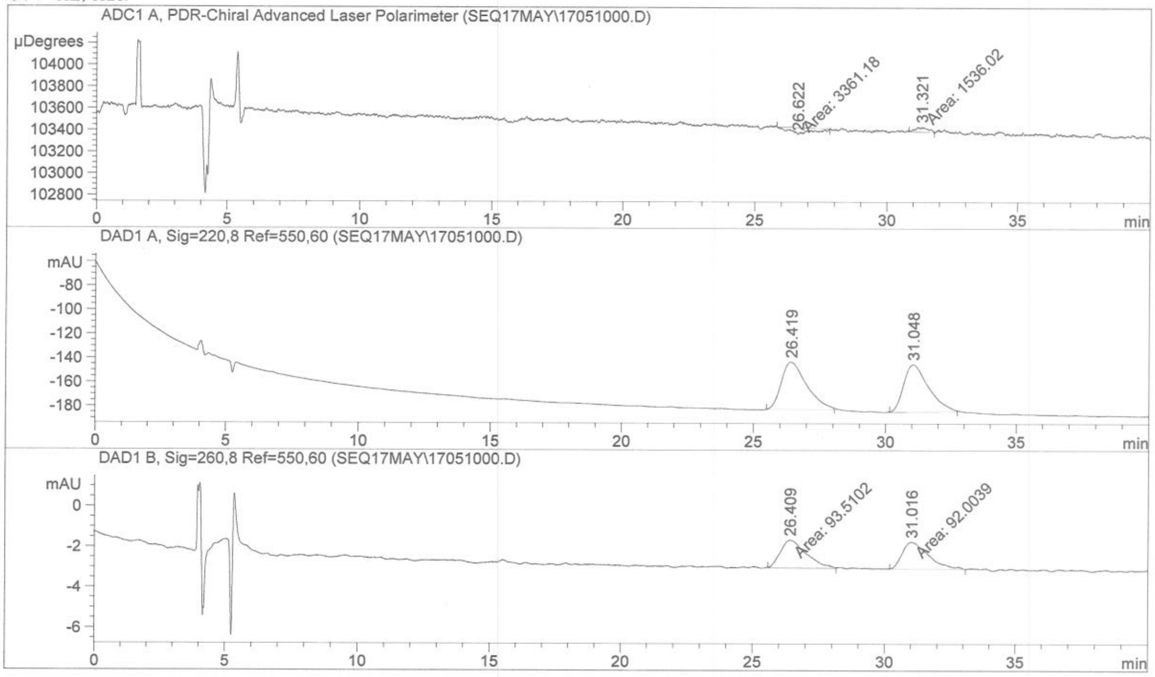


```
=====================================================================
                       Area Percent Report
=====================================================================

Sorted By             :      Signal
Multiplier            :      1.0000
Dilution              :      1.0000
Use Multiplier & Dilution Factor with ISTDs


Signal 1: ADC1 A, PDR-Chiral Advanced Laser Polarimeter

Peak RetTime Type  Width     Area      Height     Area
  #   [min]        [min]  µDegrees*  µDegrees      %
----|-------|----|-------|----------|----------|--------|
   1  26.622 MP N  0.8638 3361.17749   64.85577  68.6347
   2  31.321 MM    0.5413 1536.01880   47.29240  31.3653

Totals :                  4897.19629  112.14817

 Results obtained with enhanced integrator!
```

PDR 5/18/2010 11:41:21 AM EEN

VISITING ADDRESS | POSTAL ADDRESS | TELEPHONE | FAX | E-MAIL | TRADE REGISTER | INTERNET
Kadijk 3, 9747 AT Groningen, The Netherlands | P.O. Box 2253, 9704 CE Groningen | +31(0)505757272 | +31(0)505757399 | info@syncom.nl | 020 411 05 | www.syncom.nl

Designing chemistry

Chiral HPLC separation praziquanamine enantiomers

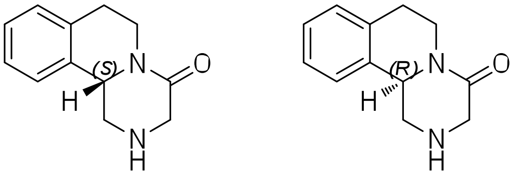

(S)-(+) (R)-(-)

Data File D:\DATA\SEQ17MAY\17051000.D Sample Name: JSN147927-RAC

Signal 2: DAD1 A, Sig=220,8 Ref=550,60

Peak #	RetTime [min]	Type	Width [min]	Area [mAU*s]	Height [mAU]	Area %
1	26.419	BB	0.9100	2640.84326	39.49873	50.1862
2	31.048	BB	0.9498	2621.25171	39.31960	49.8138

Totals : 5262.09497 78.81834

Results obtained with enhanced integrator!

Signal 3: DAD1 B, Sig=260,8 Ref=550,60

Peak #	RetTime [min]	Type	Width [min]	Area [mAU*s]	Height [mAU]	Area %
1	26.409	MM	1.1353	93.51015	1.37283	50.4060
2	31.016	MM	1.1587	92.00387	1.32334	49.5940

Totals : 185.51402 2.69617

Results obtained with enhanced integrator!

*** End of Report ***

VISITING ADDRESS POSTAL ADDRESS TELEPHONE FAX E-MAIL TRADE REGISTER INTERNET

Kadijk 3, 9747 AT Groningen, The Netherlands P.O. Box 2253, 9704 CE Groningen +31(0)505757272 +31(0)505757399 info@syncom.nl 020 411 05 www.syncom.nl

SYNCOM

Designing chemistry

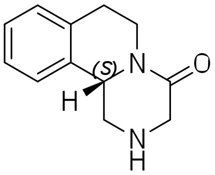

(S)-(+)

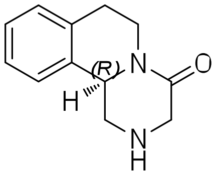

(R)-(-)

Chiral HPLC separation praziquanamine enantiomers

Data File D:\DATA\SEQ17MAY\17051001.D   Sample Name: JSN147927-RAC

===

```
Injection Date   : 5/17/2010 3:26:27 PM          Seq. Line :    2
Sample Name      : JSN147927-RAC                  Location : Vial 21
Acq. Operator    : JGR                                 Inj :    1
                                                Inj Volume : 4 µl
Method           : C:\HPCHEM\1\METHODS\C2ETOH-N.M
Last changed     : 5/17/2010 2:29:49 PM by EEN
Chiralpak AS-H
Heptane/EtOH 80:20
0.5 ml/miN
```

AS-H

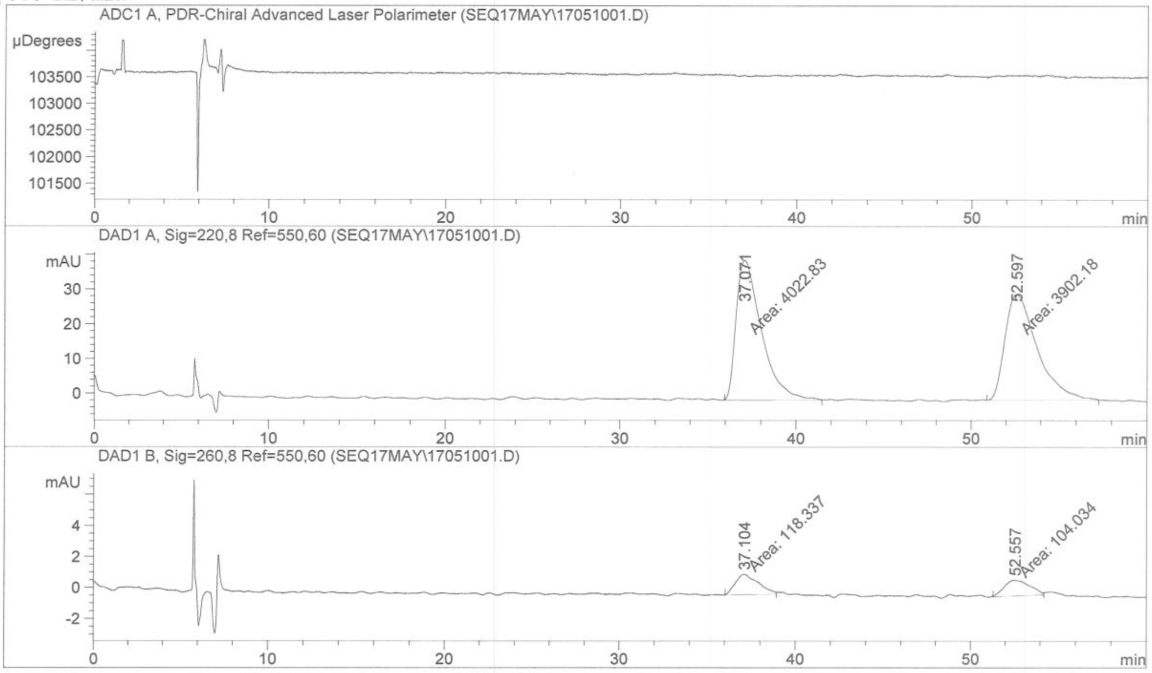

===

Area Percent Report

===

```
Sorted By              :      Signal
Multiplier             :      1.0000
Dilution               :      1.0000
Use Multiplier & Dilution Factor with ISTDs
```

Signal 1: ADC1 A, PDR-Chiral Advanced Laser Polarimeter

Signal 2: DAD1 A, Sig=220,8 Ref=550,60

Peak #	RetTime [min]	Type	Width [min]	Area [mAU*s]	Height [mAU]	Area %
1	37.071	MM	1.6626	4022.82568	40.32782	50.7612
2	52.597	MM	2.1324	3902.17676	30.49917	49.2388

Totals : 7925.00244 70.82700

Results obtained with enhanced integrator!

VISITING ADDRESS POSTAL ADDRESS TELEPHONE FAX E-MAIL TRADE REGISTER INTERNET

Kadijk 3, 9747 AT Groningen, The Netherlands P.O. Box 2253, 9704 CE Groningen +31(0)505757272 +31(0)505757399 info@syncom.nl 020 411 05 www.syncom.nl

SYNCOM

Designing chemistry

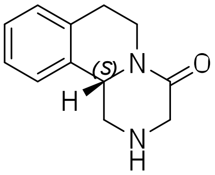

(S)-(+)

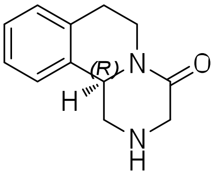

(R)-(-)

Chiral HPLC separation praziquanamine enantiomers

Data File D:\DATA\SEQ17MAY\17051001.D

Sample Name: JSN147927-RAC

Signal 3: DAD1 B, Sig=260,8 Ref=550,60

Peak #	RetTime [min]	Type	Width [min]	Area [mAU*s]	Height [mAU]	Area %
1	37.104	MM	1.4930	118.33729	1.32101	53.2161
2	52.557	MM	1.6920	104.03395	1.02474	46.7839

Totals : 222.37124 2.34575

Results obtained with enhanced integrator!

*** End of Report ***

VISITING ADDRESS POSTAL ADDRESS TELEPHONE FAX E-MAIL TRADE REGISTER INTERNET

Kadijk 3, 9747 AT Groningen, The Netherlands P.O. Box 2253, 9704 CE Groningen +31(0)505757272 +31(0)505757399 info@syncom.nl 020 411 05 www.syncom.nl

Chiral HPLC separation praziquanamine enantiomers

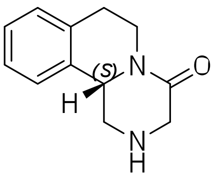

(S)-(+)

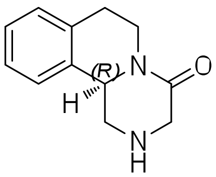

(R)-(-)

===

Injection Date : 5/17/2010 6:55:51 PM Seq. Line : 3
Sample Name : JSN147927-RAC Location : Vial 21
Acq. Operator : JGR Inj : 1
Inj Volume : 4 µl

IB

Method : C:\HPCHEM\1\METHODS\C5ETOH-N.M
Last changed : 5/17/2010 4:33:43 PM by EEN
Chiralpak IB
Heptane/EtOH 60/40
0.7 ml/min

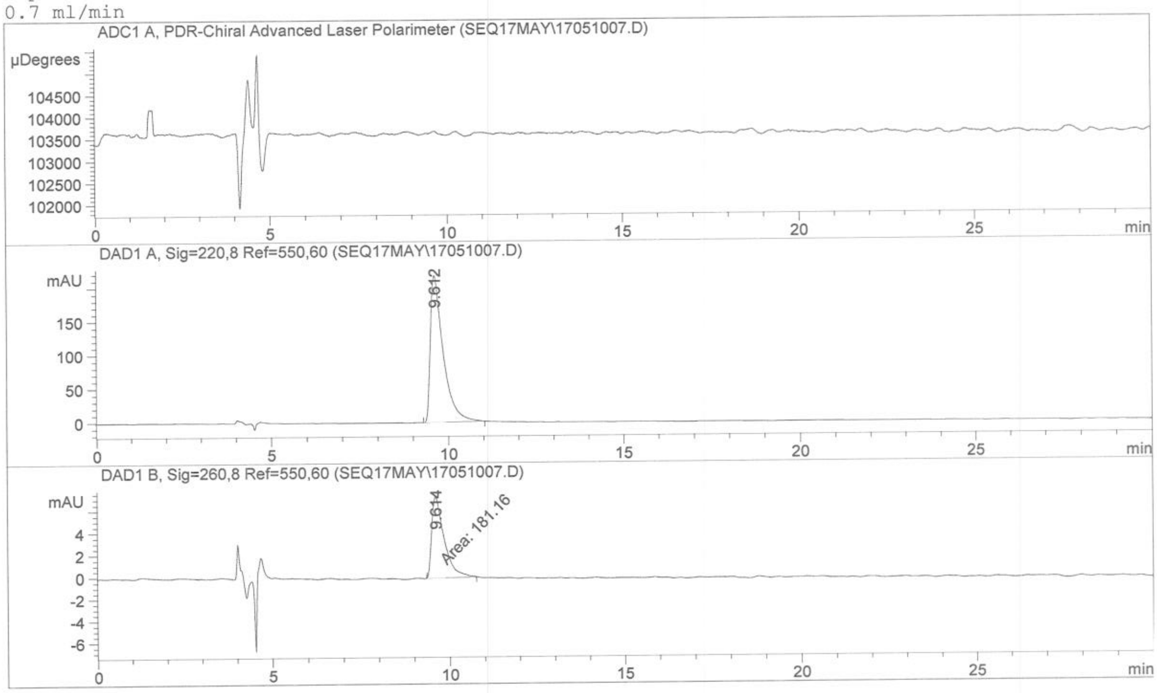

===

Area Percent Report

===

Sorted By : Signal
Multiplier : 1.0000
Dilution : 1.0000
Use Multiplier & Dilution Factor with ISTDs

Signal 1: ADC1 A, PDR-Chiral Advanced Laser Polarimeter

Signal 2: DAD1 A, Sig=220,8 Ref=550,60

Peak #	RetTime [min]	Type	Width [min]	Area [mAU*s]	Height [mAU]	Area %
1	9.612	PB	0.3668	5476.71533	218.22020	100.0000

Totals : 5476.71533 218.22020

Results obtained with enhanced integrator!

VISITING ADDRESS POSTAL ADDRESS TELEPHONE FAX E-MAIL TRADE REGISTER INTERNET

Kadijk 3, 9747 AT Groningen, The Netherlands P.O. Box 2253, 9704 CE Groningen +31(0)505757272 +31(0)505757399 info@syncom.nl 020 411 05 www.syncom.nl

SYNCOM

Designing chemistry

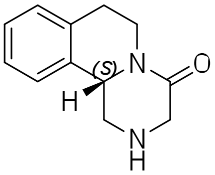

(S)-(+)

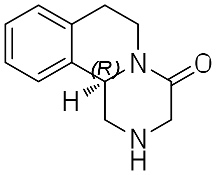

(R)-(-)

Chiral HPLC separation praziquanamine enantiomers

Data File D:\DATA\SEQ17MAY\17051007.D

Sample Name: JSN147927-RAC

Signal 3: DAD1 B, Sig=260,8 Ref=550,60

Peak #	RetTime [min]	Type	Width [min]	Area [mAU*s]	Height [mAU]	Area %
1	9.614	MM	0.4107	181.16000	7.35195	100.0000

Totals : 181.16000 7.35195

Results obtained with enhanced integrator!

*** End of Report ***

VISITING ADDRESS POSTAL ADDRESS TELEPHONE FAX E-MAIL TRADE REGISTER INTERNET

Kadijk 3, 9747 AT Groningen, The Netherlands P.O. Box 2253, 9704 CE Groningen +31(0)505757272 +31(0)505757399 info@syncom.nl 020 411 05 www.syncom.nl

Designing chemistry

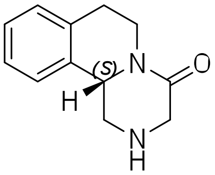

(S)-(+)

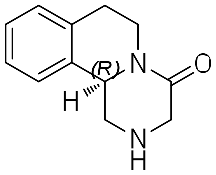

(R)-(-)

Chiral HPLC separation praziquanamine enantiomers

Data File D:\DATA\SEQ17MAY\17051005.D Sample Name: JSN147927-RAC

```
=====================================================================
Injection Date  : 5/17/2010 4:51:14 PM          Seq. Line :    1
Sample Name     : JSN147927-RAC                  Location : Vial 21
Acq. Operator   : JGR                                 Inj :    1
                                               Inj Volume : 4 µl
Acq. Method     : C:\HPCHEM\1\METHODS\C3ETOH-N.M
Last changed    : 5/17/2010 4:49:42 PM by JGR
                  (modified after loading)
Analysis Method : C:\HPCHEM\1\METHODS\C3ETOH-N.M
Last changed    : 5/18/2010 11:44:18 AM by EEN
                  (modified after loading)
chiralcel OD-H
Heptane/EtOH 60/40
0.5 ml/min
```

OD-H

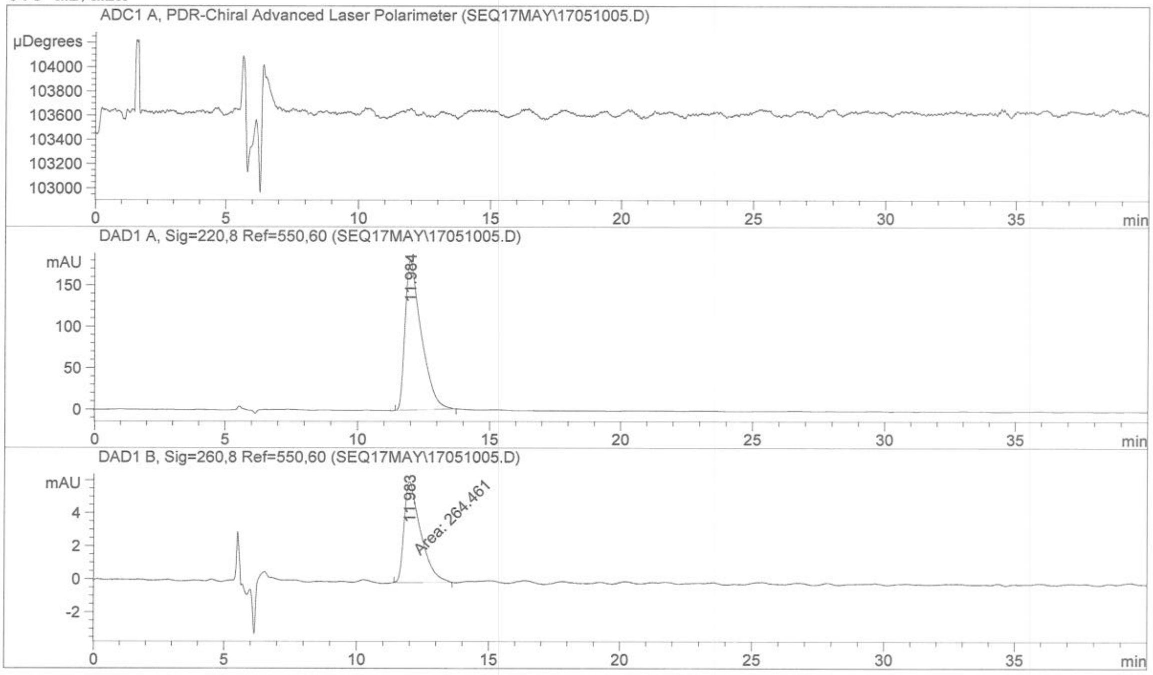


```
=====================================================================
                     Area Percent Report
=====================================================================

Sorted By             :      Signal
Multiplier            :      1.0000
Dilution              :      1.0000
Use Multiplier & Dilution Factor with ISTDs


Signal 1: ADC1 A, PDR-Chiral Advanced Laser Polarimeter


Signal 2: DAD1 A, Sig=220,8 Ref=550,60

Peak RetTime Type  Width     Area      Height     Area
  #   [min]        [min]   [mAU*s]     [mAU]        %
----|-------|----|-------|----------|----------|--------|
   1  11.984 BB    0.6056 7700.32471  180.17667 100.0000

Totals :                  7700.32471  180.17667
```


VISITING ADDRESS POSTAL ADDRESS TELEPHONE FAX E-MAIL TRADE REGISTER INTERNET

Kadijk 3, 9747 AT Groningen, The Netherlands P.O. Box 2253, 9704 CE Groningen +31(0)505757272 +31(0)505757399 info@syncom.nl 020 411 05 www.syncom.nl

SYNCOM

Designing chemistry

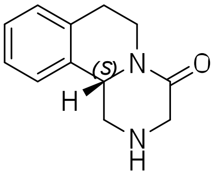

(S)-(+)

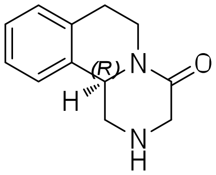

(R)-(-)

Chiral HPLC separation praziquanamine enantiomers

Data File D:\DATA\SEQ17MAY\17051005.D

Sample Name: JSN147927-RAC

Results obtained with enhanced integrator!

Signal 3: DAD1 B, Sig=260,8 Ref=550,60

Peak #	RetTime [min]	Type	Width [min]	Area [mAU*s]	Height [mAU]	Area %
1	11.983	MM	0.7218	264.46082	6.10646	100.0000

Totals : 264.46082 6.10646

Results obtained with enhanced integrator!

*** End of Report ***
