# Supplementary material for: Resolution of Praziquantel
Source: PLoS Negl Trop Dis. 2011 Sep 20;5(9):e1260. doi: 10.1371/journal.pntd.0001260 (PMC3176743; doi:10.1371/journal.pntd.0001260)
Supplement: Text S2 — Contributors to The Synaptic Leap project. (DOCX) [file pntd.0001260.s015.docx]

Contributors to the Synaptic Leap Project concerning the Resolution of Praziquantel

Ginger Taylor (Workday, and Founder, The Synaptic Leap)

Jean-Paul Seerden (Syncom B.V.)

Nick Tyrell (Almac Sciences)

William Jackson (Creative Chemistry)

Rob Bryant (Development Chemicals)

Ryan Pakula (formerly Harvey Mudd College, visitor to The University of Sydney)

Clarke Slemon (a.k.a. kilomentor)

Craig Williams (University of Queensland)

Jean-Claude Bradley (Drexel University)

Cameron Neylon (STFC)

Ahamed Muneer (University of Sydney)

Thomas Webb (St. Jude Children’s Research Hospital)

Tinopiwa Goronga (St. Jude Children’s Research Hospital)

Bobby Baum

Marvin S. Yu

Heiko Schill

Graham Steel

Wayne Best (Epichem, Ltd)

Marc Marti-Renom

“Mike”

“Paul”

“Barry”

“Quintus”

“Guest” x 2

Others contributed via the Custom Organic Synthesis and Process Chemistry group at LinkedIn (LinkedIn Website. Available: <http://www.linkedin.com/groups?home=&gid=1061737&trk=anet_ug_hm>. Accessed 2011 Jun 27), as well as by private email contact (to MHT).
